# MiR-1976/NCAPH/P65 axis inhibits the malignant phenotypes of lung adenocarcinoma

**DOI:** 10.1038/s41598-024-61261-6

**Published:** 2024-05-16

**Authors:** Peiluo Huang, Hongtao Zhao, Ruonan Sun, Chunyan Liu, Lei Wu, Yao Wang, Zhengwei Gan, Xiuzhen Yang, Juan Du

**Affiliations:** 1 Department of Immunology, College of Basic Medicine, Guilin Medical University, Guilin, 541199 Guangxi China; 2 College of Pharmacy, Guilin Medical University, Guilin, 541199 Guangxi China; 3 Central Laboratory, The First Affiliated Hospital of Shandong First Medical University, Shandong Provincial Qianfoshan Hospital, Jinan, 250014 Shandong China; 4 College of Department of Information & Library Science, Guilin Medical University, Guilin, 541004 China; 5 School of Clinical Medicine, Guilin Medical University, Guilin, 541199 Guangxi China; 6 Department of Clinical Laboratory, Zibo Central Hospital, 54 Gongqingtuan Xi Road, Zibo, 255036 China

**Keywords:** Non-small-cell lung cancer, Oncogenes, Cancer genomics

## Abstract

Lung adenocarcinoma (LUAD) is a malignancy with an abysmal survival rate. High metastasis is the leading cause of the low survival rate of LUAD. NCAPH, an oncogene, is involved in the carcinogenesis of LUAD. However, the regulation of NCAPH in LUAD remains controversial. In this work, we identified an up-regulation of NCAPH in LUAD tissues. Patients who expressed more NCAPH had shorter overall survival (OS). Furthermore, NCAPH overexpression promoted LUAD cell migration while inhibiting apoptosis. MiR-1976 and miR-133b were predicted to target NCAPH expression by searching TargetScan and linkedomics databases. Following that, we confirmed that miR-1976 suppressed NCAPH by directly targeting a 7-bp region of NCAPH 3′ untranslated regions (UTR). In addition, increased expression of miR-1976 decreased the proliferation & migration and promoted apoptosis of LUAD cells, and the re-introduction of NCAPH reversed these influences. Furthermore, the xenograft and metastasis mouse models also confirmed that miR-1976 inhibited tumor growth and metastasis in vivo by targeting NCAPH. Finally, we found that MiR-1976 targeting NCAPH blocked the activation of NF-κB. In conclusion, miR-1976 inhibits NCAPH activity in LUAD and acts as a tumor suppressor. The miR-1976/NCAPH/NF-κB axis may, in the future, represent crucial diagnostic and prognostic biomarkers and promising therapeutic options.

Nearly one-fourth of all cancer-related fatalities worldwide are caused by lung carcinoma, one of the most common forms of cancer^1^. Because lung cancer is highly destructive and metastatic, only a tiny percentage of patients survive five years after diagnosis^2^. The two histological lung cancer types are small and non-small cell lung cancers (SCLC and NSCLC, respectively). The most typical kind of lung cancer, NSCLC, accounts for around 80% of cases and more than 50% of lung cancer cases are lung adenocarcinoma (LUAD), the most common histological subtype of NSCLC^3,4^. The identification of LUAD-regulating molecules is crucial for early detection and focused treatment.

NCAPH, Non-SMC Condensin I complex subunit H, was first found in Drosophila by Cabello et al. in 1997. The gene of NCAPH is located in chromosome 2q11.2^5^. Alberto Viera et al. determined that NCAPH was located in the chromosome axis and enriched at the ends of centromeres and telomeres^6^. NCAPH was linked to oncogenesis and tumor growth in several studies^7–9^. In breast cancer^7^, endometrial cancer^8^, colon cancer^10^, prostate cancer^11^, and hepatocellular carcinoma^12^, NCAPH was highly expressed as a tumor promoter. However, the potential role of NCAPH in the etiology of many cancers, particularly LUAD, remains debatable.

MicroRNAs, called small non-coding RNA molecules, can control gene expression and act as tumor suppressors or oncogenes, affecting tumor cell development and death. Approximately 60% of human mRNA that codes for proteins is controlled by the 2588 mature miRNAs in the human genome^13^. Additionally, miRNA-based gene therapy offers a different and alluring approach to suppressing cancer^14,15^. Through targeting the SNAIL gene, MiR-34 interfered with the progression of tumors during the epithelial-mesenchymal transition (EMT). MiR-34a was a viable target for cancer treatment because it is a well-researched tumor suppressor^16^. MiR-199a/b-3p targeted the carcinogenic gene NCAPH, which is implicated in the development of prostate cancer^17^. TRPM8 and other oncogenes shared targets of miR-126-3p and miR-126-5p in LUAD^18^. Additionally, in glioma and hepatocellular carcinoma, miR-133b could directly target Sirt1 and decrease cell expansion and invasion^19,20^. MiR-1976 exhibited reduced expression in breast, colorectal, and NSCLC cancers and was associated with a worse prognosis for cancer patients^21–23^.

We report here that miR-1976 expression was down-regulated in LUAD tissue and was inversely related to NCAPH mRNA expression. Moreover, patients with higher miR-1976 expression have more prolonged OS. In vitro and in vivo, miR-1976 impairs cell growth and metastasis and promotes apoptosis of lung adenocarcinoma by targeting NCAPH/ NF-κB axis.

## Methods

### Examination of the TCGA and GEO databases using bioinformatics

Five hundred and fifteen LUAD specimens and 59 normal tissue data were extracted from the TCGA database (https://cancergenome.nih.gov). The Gene Expression Synthesis (GEO database, available at https://www.ncbi.nlm.nih.gov/geo/) contains GSE10072 and GSE43458. TargetScan (https://www.targetscan.org/) projected 1497 predicted miRNAs to be connected with NCAPH, and 130 miRNAs were found to be negatively correlated with NCAPH using the Linkedomics database (http://linkedomics.org/login.php).

### Human tissue specimens

The Zibo Central Hospital provided 14 pairs of tumor tissues and nearby tissues. Before surgery, all patients had not received any other form of treatment, such as chemotherapy or radiotherapy. All patients were informed and consented to the study before the study. In addition, this study has been approved by the Medical Ethics Expert Committee of Zibo Central Hospital (No. 201912011) and the Ethics Committee of Guilin Medical University (GLMC20200614). We confirmed that all methods are carried out in accordance with the relevant guidelines and regulations.

### Western blot analysis

Cell and tissue samples were homogenized using a protease inhibitor-containing immunoprecipitation lysis solution ( Beyotime, Shanghai, China ). The corresponding protein was isolated by 10% SDS-PAGE Gel. During imprinting, the cutting is performed prior to hybridization with the antibody. The antibodies were applied as follows: NCAPH goat polyclonal antibody (dilution: 1: 1000; category number NBP1-88345; Novus Biologicals), NF-κB p65 (dilution: 1: 1000; classification number D14E12; Cell Signaling Technology), Phospho-NF-kB p65 (Ser536) Antibody(dilution: 1: 1000; classification number AF2006; Affinity) and GAPDH was cultured overnight in the presence of internal reference antibodies (dilution, 1: 1000; classification number D16H11; Cell Signaling Technology). Proteins can be identified using immunoreaction strips and the Western Lightning chemiluminescence reagent from Millipore (Darmstadtmerk KGaA, Germany). Additionally, the bands were examined using ImageJ (version 1.8; National Institute of Health, Bethesda, Maryland, USA).

### Real-time PCR analysis

Total RNA was extracted from cells using a complete kit for RNA extraction (Tiangen Biotech, Beijing, China). The PrimeScript RT kit (TaKaRa, Japan) reverse-transcribes total RNA into first-strand complementary DNA. Real-time PCR was carried out on a Bio-Rad iQ5 machine using the SYBR Green PCR Kit (TaKaRa, Japan). The primers for NCAPH and β-actin are as follows : NCAPH: Forward,5’-ACAGTGCCTCCTCTCCTTCA-3’, Reverse,5’-CCGCTCCTTCTCATCGTCAT-3’. Each sample was tested in triplicate. The relative expression levels of each gene were calculated using the 2-ΔΔCt method.

### Cell transfection

Created the NCAPH overexpressed lentivirus (Lv-NCAPH) and NCAPH gene knockout lentivirus (sh-NCAPH) were purchased from Genomeditech (Shanghai, China) and GeneChem (Shanghai, China), respectively. The A549 and NCI-H1976 cells were implanted into the 12-well plate, and the related lentiviruses were added to the wells. MiR-1976 / 133b (GenePharma, Shanghai, China) provided the mimics, inhibitors, and scrambled negative control RNA. In the six-well plate, A549 and NCI-H1976 cells were implanted. Lipofectamine 2000 (Invitrogen) transfected cells with miRNA mimic (200 pmol), inhibitor, or negative control. GeneChem (Shanghai, China) created the miR-1976 lentivirus, which was used in vivo for Xenograft and metastasis assays.

### Luciferase reporter assay

The three predicted miR-1976 target locations of NCAPH 3’UTR were cloned into XbaI restriction enzyme cutting site of luciferase reporter plasmid pGL3-promoter and constructed pGL3-T1 (3'UTR: binding site: 23-29nt, position 1 to 68nt), pGL3-T2 (3'UTR: binding site: 1627-1633nt, position1543 to 1651nt), pGL3-T3 (3'UTR: binding site: 2889- 2896nt, position 2857 to 2922nt). The pGL3-T2 mut plasmid was constructed bearing the mutating 3'UTR:1627-1633nt (GCAGGAG mutation to TACTTCT). The A549 cells were co-transfected with 400 ng of pGL3-T1, pGL3-T2, and pGL3-T3 in 48-well culture plates and 50 pmol miR-1976 mimics, miR-1976 inhibitors, or corresponding negative control microRNA. Luciferase activity was normalized to the internal control pRL-TK. Using a luciferase assay instrument (Madison Promega, Wisconsin, USA), luciferase analysis was carried out after 48 h.

### Cell migration assay

The suspension cells (3 × 10^4^) in serum-free DMEM were infused into Transwell chambers. The Transwell chamber was cleaned with PBS after 48 h, fixed for 20 min with methanol, stained with 0.5% crystal violet, rinsed for 20 min with PBS to clean the background, dried, and photographed after sealing.

### Cell apoptosis and cell cycle assay

The trypsinized A549/NCI-H1975 cells were adjusted to 3 × 10^5^ cells/mL with serum-containing DMEM/RPMI-1640 medium. The Annexin V-APC and the 7-AAD Apoptosis Detection Kit (MultiSciences, China) were used in the cell apoptosis assay. The A549 or NCI-H1975 cells were transfected with miR-1976 mimic, miR-1976 inhibitor, Lv-NCAPH lentivirus, or sh-NCAPH lentivirus for 24 h. Then, the cells were treated with serum-free DMEM/RPMI-1640 medium for 24 h to induce apoptosis. Next, the cells were stained with Annexin V-APC and 7-AAD for 30 min in the dark. Cell cycle detection was performed using cell cycle staining buffer (MultiSciences, China). Cell cycle detection was conducted when the treated cells were washed with PBS and then stained with cell cycle staining buffer for 30 min. The samples were finally tested using a flow cytometer(Becton Dickinson), and the apoptosis populations were calculated by FlowJo X software. Similarly, the cell cycle was analyzed using FlowJo X software.

### Laser confocal

A cell slide was first laid on a 12-well cell culture plate, and 1 × 10^5^ cells/holes were inoculated. After 24 h of culture, the cells on the slide were stained with NF-κB activation-nuclear transport detection kit (Beytime, China), and then the slide was sealed on pathological tissue slides. The image was captured using a laser scanning microscope (Zeiss) to detect the fluorescence of DAPI and Cy3 under a 40-x oil lens.

### CCK8 assay

In 96-well plates, LUAD cell groups were seeded. Each 96-well plate well was seeded with 500 cells per 100 μL. Replaced with fresh medium, ten microliters of CCK8 reagent were added to each well. Absorbance was measured at 450 nm in each 96-well plate after 24, 48, 72, and 96 h.

### Xenograft assay and metastasis assay in vivo

The Experimental Animal Ethics Committee of Guilin Medical university has reviewed and approved all animal experiments (No. GLMC-IACUC-2023005). This study is reported in accordance with ARRIVE guidelines. Animal Technology Vital River Lab (Beijing, China) supplied male BALB/c nude mice. In vivo xenograft assay, 1 × 10^7^ A549 cells stably transfected with control lentivirus, miR-1976 lentivirus, Lv-NCAPH lentivirus, miR-1976 lentivirus + Lv-NCAPH lentivirus were subcutaneously injected into mice, respectively. Naked mice were euthanized under anesthesia after four weeks of injection. The tumor was removed and weighed. In the metastatic assay, naked mice were injected with 1 × 10^7^ A549 cells. After four weeks of injection, these mice were euthanized under anesthesia, and the lungs were removed and dyed using Bouin's solution. For euthanasia, each animal was euthanized with an overdose of sodium pentobarbital. We confirmed that all methods are carried out in accordance with the relevant guidelines and regulations.

### Statistical analysis

The statistical analyses used GraphPad Prism 8 (GraphPad Software Inc., San Diego, CA, USA) and SPSS v.25.0 (SPSS Inc., Chicago, IL, USA). The t-test was utilized to compare the two groups. One-way ANOVA was used to compare more than two groups. Correlations between clinical pathological variables (age, gender, TNM stages, T stage, N stage, M stage, and anatomic location) and NCAPH expression were assessed using Fisher's exact and Mann-Whitney U tests. P values were always two-sided, and a statistically significant P = 0.05 was used. The Kaplan-Meier with Log-rank (Mantel-Cox) test was utilized to calculate a survival analysis.

## Results

### NCAPH is up-regulated in LUAD and associated with poor prognosis in patients with LUAD

**Figure 1:**
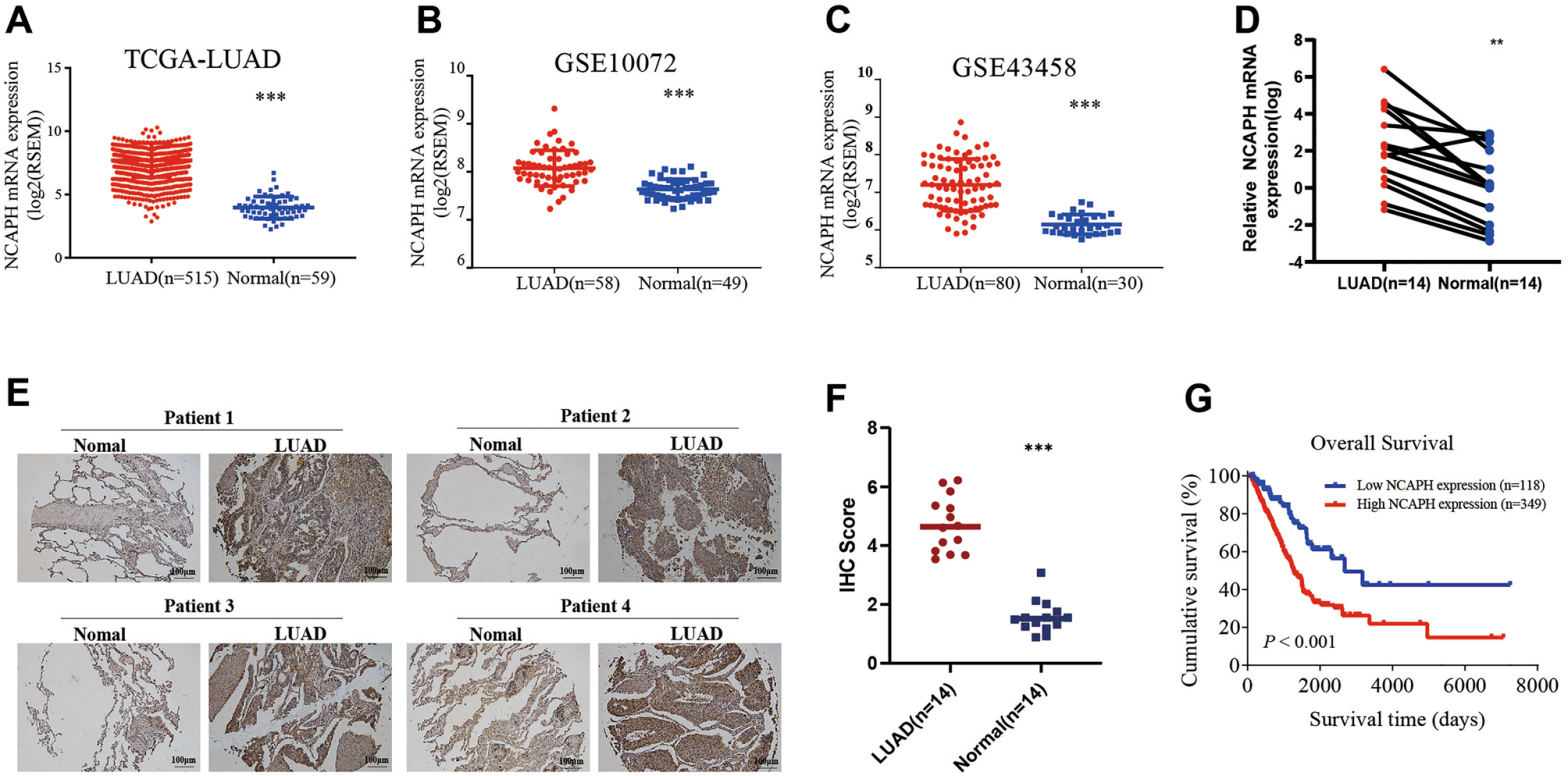
NCAPH is up-regulated in LUAD and is associated with poor prognosis in patients with LUAD. Analysis of NCAPH mRNA expression in the TCGA dataset (**A**), GEO dataset GSE10072 (**B**), and dataset GSE43458 (**C**). NCAPH mRNA expression was detected in 14 paired human LUAD samples (**D**). IHC was used to detect NCAPH expression in LUAD tissues and adjacent normal tissues (P < 0.001) (**E**, **F**). In the TCGA database, Kaplan–Meier survival analysis was used to determine the prognostic significance of NCAPH expression (**G**). * *P* < 0.05, **P < 0.01, ***P < 0.001.

To identify the potential biomarkers for LUAD, we first searched RNA-seq datasets from the GEO and the TCGA database. The TCGA data indicated that the level of NCAPH mRNA in LUAD tissues was significantly higher than that in normal tissues (Fig. 1A, P < 0.001). Similarly, GEO dataset GSE10072 (Fig. 1B, P < 0.001) and GEO dataset GSE43458 (Fig. 1C, P < 0.001) also showed NCAPH mRNA was up-regulated in LUAD samples as compared to normal controls. We further detected NCAPH mRNA levels in 14 LUAD and adjacent standard lung specimen pairs. We confirmed that the NCAPH mRNA level was significantly higher in LUAD tissues compared to those of the noncancerous counterparts (Fig. 1D, P < 0.001). In the 14 pairs of LUAD tissues, NCAPH expression was detected by IHC, showing that the level of NCAPH in LUAD tissues was higher than in normal tissues. (Fig. 1E, P < 0.001). Furthermore, we investigated the predictive significance of NCAPH in LUAD using the TCGA dataset. Using X-tile software, LUAD patients were split into two groups: those with high expression (n = 118) and those with low expression (n = 349). Patients with high NCAPH expression had a poorer overall survival rate than those with low NCAPH expression, according to the Kaplan–Meier survival curve (HR = 0.4933, 95% CI: 0.3544–0.6866, P = 0.0005) (Fig. 1G).

### NCAPH promotes proliferation &migration and inhibits apoptosis of LUAD cells

**Figure 2:**
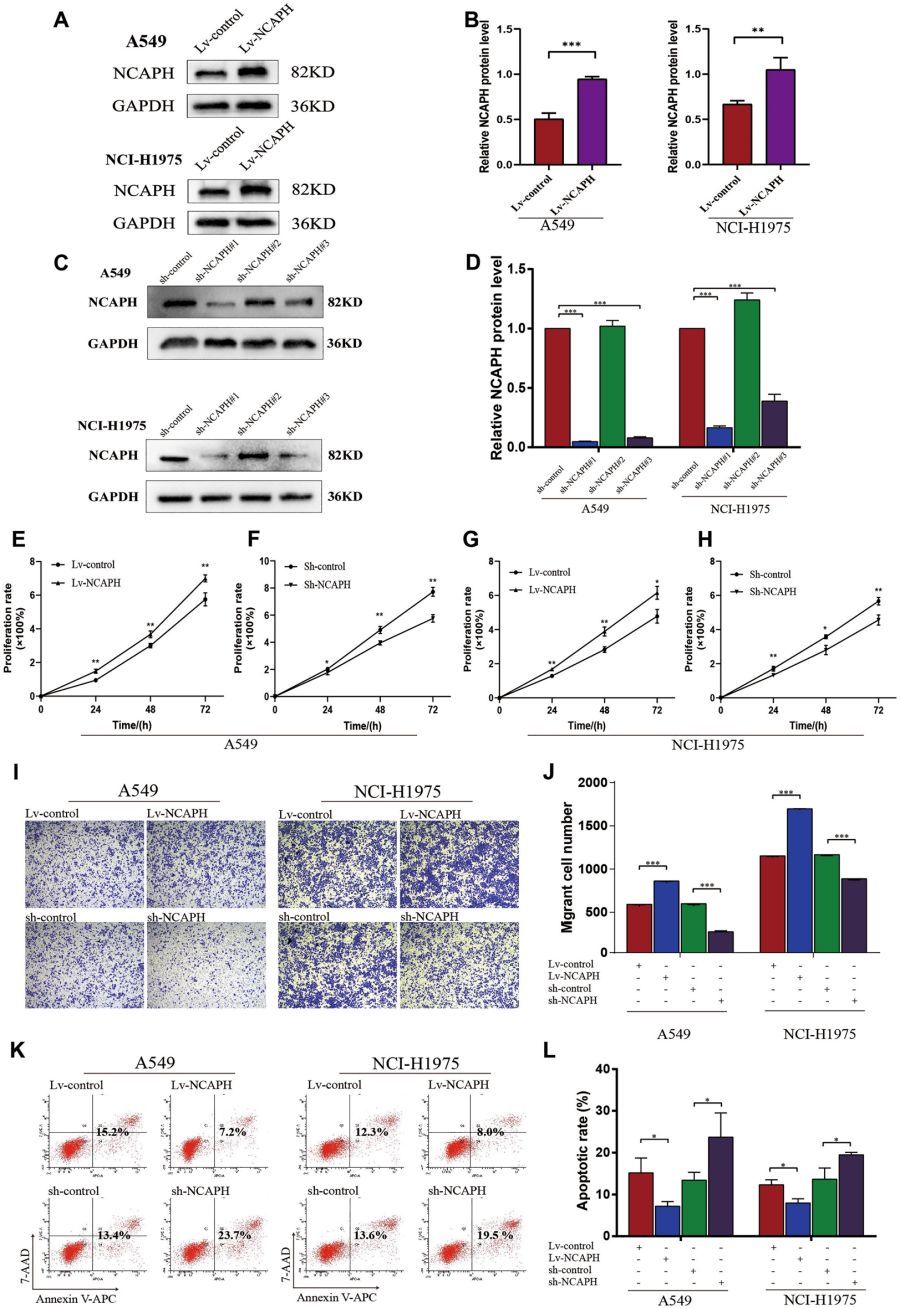
NCAPH promotes proliferation &migration and inhibits apoptosis of LUAD cells. NCAPH overexpression/knockdown lentivirus was used to treat A549 and NCI-H1975 cells. NCAPH proteins were detected by western blot assay (**A**, **C**) and quantitative analysis (**B**, **D**). The viability of LUAD cells was detected by CCK8 assay (**E**–**H**). Transwell assays were performed to detect cell migration (**I**, **J**). Apoptosis was measured by flow cytometry (**K**, **L**). * *P* < 0.05, ** *P* < 0.01, ***P < 0.001.

**Figure 3:**
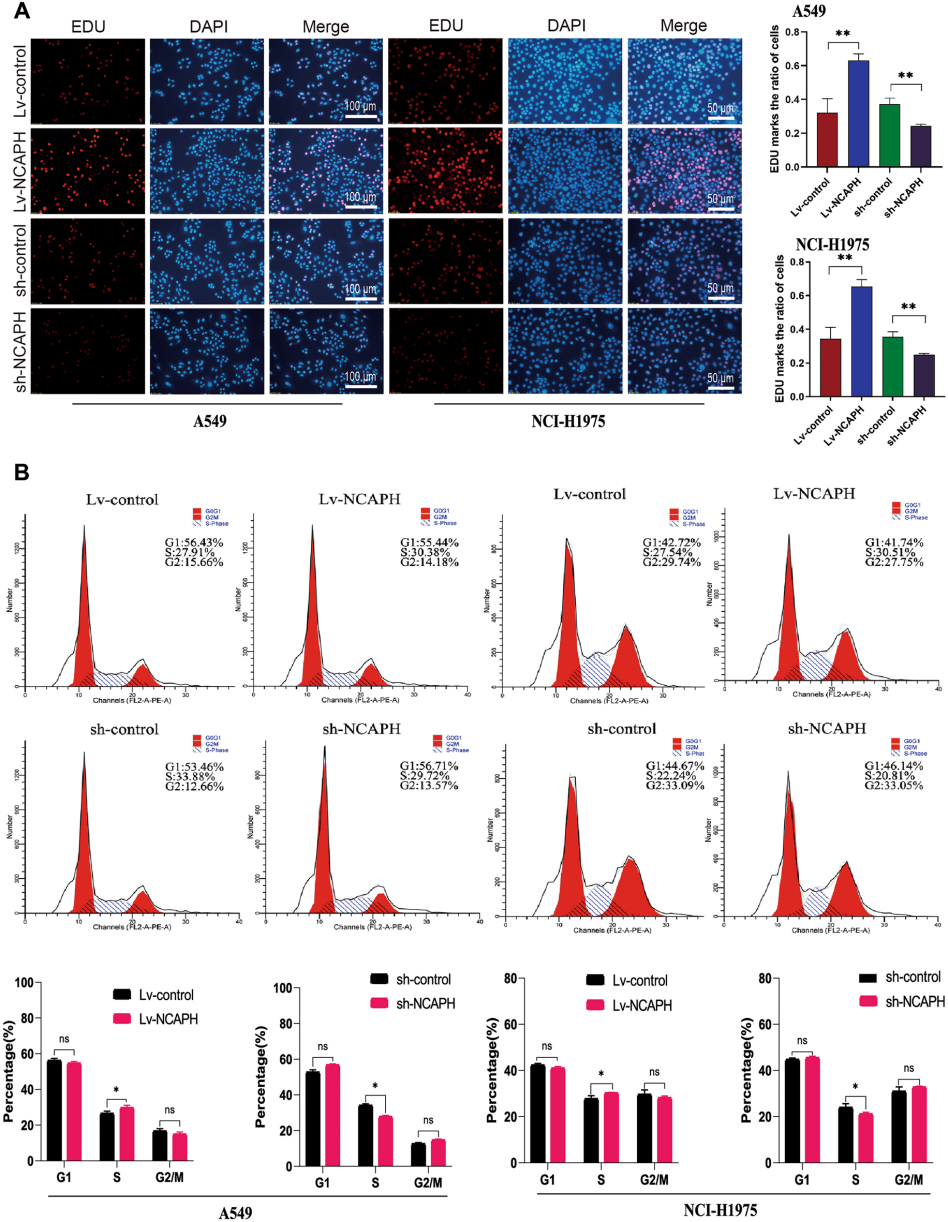
NCAPH promotes the DNA replication activity and distribution during the S phase in LUAD cells. EdU staining was used to detect DNA replication activity in LUAD cells (**A**). Flow cytometry was used to detect the distribution of LUAD cells in the cell cycle (**B**). *P < 0.05, **P < 0.01, ***P < 0.001.

Based on the findings, we investigated the effect of NCAPH on cell proliferation, migration, and apoptosis in LUAD cell lines (A549, NCI-H1975). Enhancement or silencing of NCAPH expression was performed by transfecting NCAPH knockdown/overexpression lentivirus into A549 and NCI-H1975, respectively (Fig. 2A–D). We constructed three recombinant lentiviruses directed to NCAPH and identified that sh-NCAPH#1 transfection obtained the most significant inhibitory rate for the NCAPH expression in LUAD cells (Fig. 2C,D). Therefore, sh-NCAPH#1 was used to perform the follow-up examination. The CCK8 assay revealed that Lv-NCAPH increased LUAD cells' proliferation activity compared to Lv-control cells (Fig. 2E,G, P <0.001, P < 0.05). The sh-NCAPH reduced the proliferation activity of LUAD cells (A549, NCI-H1975) compared to the sh-control (Fig. 2F,H, P <0.001, P < 0.05). NCAPH overexpression resulted in a significant enhancement in LUAD cell viability. The transwell assays showed that NCAPH overexpression promoted LUAD cell migration (Fig. 2I,J, P <0.001), and NCAPH silencing inhibited the migration in A549 and NCI-H1975 cells (Fig. 2I,J, P <0.001). Meanwhile, increased NCAPH inhibited LUAD cell apoptosis, while decreased NCAPH had the opposite effect (Fig. 2K,L, P < 0.05). On the other hand, the EdU staining found that Lv-NCAPH increased DNA replication activity in LUAD cells compared to Lv-control cells. The sh-NCAPH decreased the DNA replication activity of LUAD cells compared with sh-control (Fig. 3A, P < 0.01). Flow cytometry showed that Lv-NCAPH increased the distribution of S-phase LUAD cells compared with Lv-control cells. The sh-NCAPH decreased the distribution of S-phase LUAD cells compared with sh-control (Fig. 3B, P < 0.05).

### MiR-1976 targets NCAPH expression

**Figure 4:**
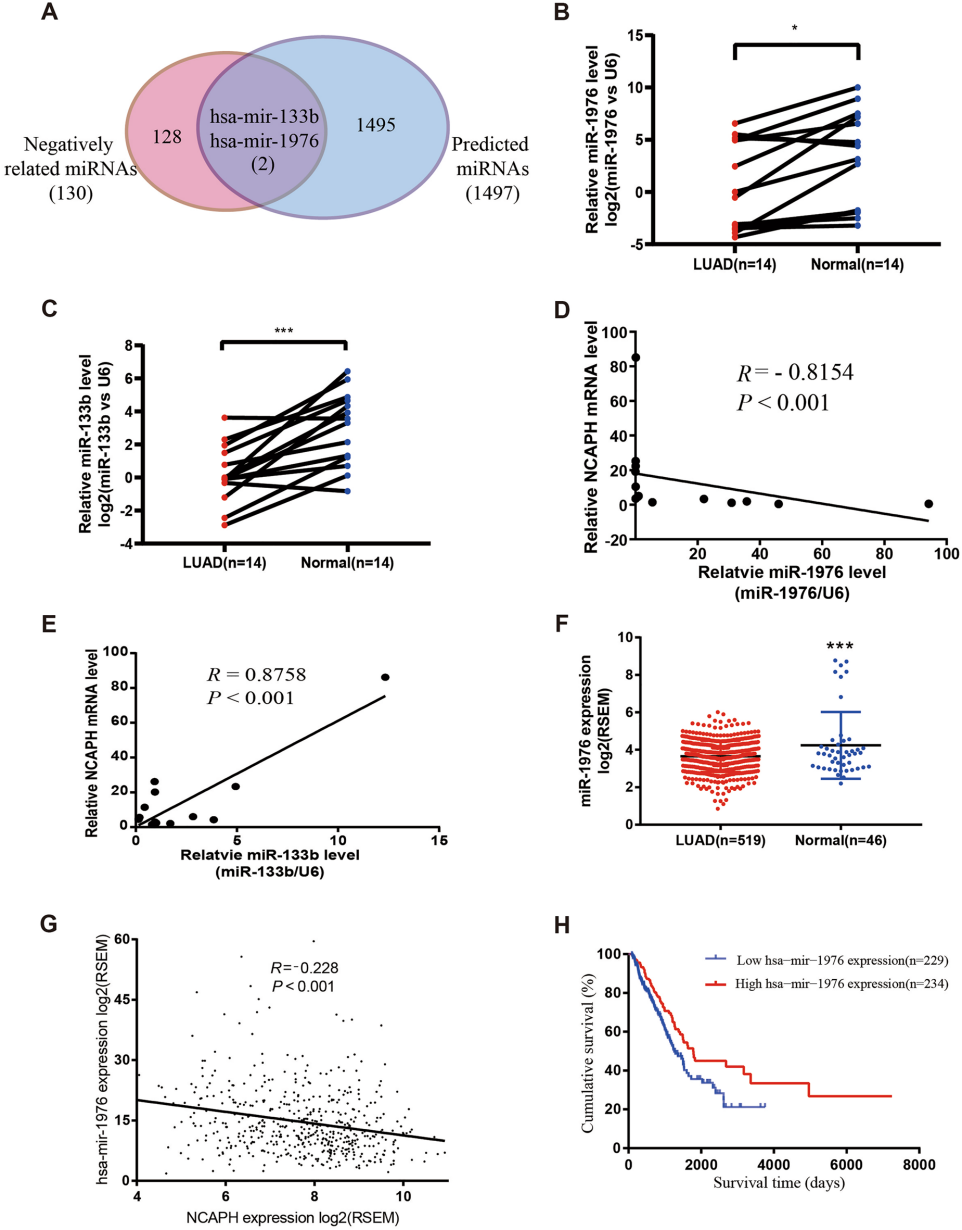
MiR-1976 targets NCAPH expression. MiR-1976 and miR-133b targeting NCAPH were identified using the TargetScan and linkedomics databases (**A**). The levels of miR-1976 and miR-133b were determined using real-time PCR in 14 pairs of clinical tissues (**B**, **C**). In 14 human LUAD tissues, the connections between miR-1976 levels and NCAPH mRNA levels (**D**) and between miR-133b levels and NCAPH mRNA levels(**E**) were examined using Pearson's correlation. The TCGA data for miR-1976 levels in LUAD and normal tissues were examined (**F**). Pearson's correlation was used to examine the relationship between miR-1976 levels and NCAPH mRNA levels in the TCGA database (**G**). The Kaplan–Meier was used to assess the prognostic value of miR-1976 (**H**). * *P* < 0.05, ** *P* < 0.01, ***P < 0.001.

In recent studies, oncogenes such as NCAPH can be targeted by some microRNAs to participate in tumor occurrence. We looked through two databases, TargetScan, and linkedomics, for potential microRNAs targeted NCAPH. The Venn diagram displayed that both databases predicted that miR-1976 and miR-133b could target NCAPH expression (Fig. 4A). To evaluate the potential clinical relationship between NCAPH mRNA expression and miR-1976 and miR-133b, we first validated miR-1976, miR-133b and NCAPH mRNA levels in 14 paired patient samples by real-time PCR. The findings demonstrated that LUAD tissues had lower miR-1976 and miR-133b levels than the nearby normal tissues (Fig. 4B, P < 0.05, Fig. 4C, P < 0.001). Additionally, the correlations between the two microRNAs and NCAPH were investigated using Pearson's correlation test. NCAPH mRNA levels and miR-1976 expression had a significant negative correlation. (Fig. 4D, R =  − 0.8154, P < 0.001). However, miR-133b level was positively correlated with NCAPH mRNA level (Fig. 4E, R = 0.8758, P < 0.001).

We also verified the above results in the TCGA database. We used 519 LUAD tissues and 46 normal tissues to detect the expression of miR-1976. As mentioned above, there was less miR-1976 in LUAD tissues than in healthy tissues (Fig. 4F, P < 0.001). The TCGA data also confirmed the negative correlation of miR-1976 level with NCAPH mRNA level (Fig. 4G, R =  − 0.228, P < 0.01). Additionally, using the TCGA dataset, we found that patients with high miR-1976 expression (n = 229) had significantly better prognoses than patients with low miR-1976 expression (n = 234) (Fig. 4H, HR = 1.548, 95% CI: 1.142–2.098, P = 0.0068). Higher miR-1976 expression might be related to advanced T, N, M, and TNM stages, according to further investigation of miR-1976 expression and clinicopathological characteristics in LUAD patients (Table 1).

**Table 1:** Association between the expression levels of miR-1976 and the clinicopathological features of patients with lung adenocarcinoma.

Variables	Numbers of patients		*χ*^*2*^	*P* value
	Low expression (229)	High expression (234)		
Age(years)				
≤ 65	115	107	0.936	0.333
> 65	114	127		
Gender				
Female	109	140	6.965	0.008
Male	120	94		
T stage				
1	61	94	3.105	0.002
2	134	116		
3	24	18		
4	10	6		
N stage				
0	140	167	2.216	0.027
1	51	36		
2	36	31		
3	2	0		
M stage				
0	214	228	4.247	0.039
1	15	6		
TNM stage				
I	114	142	2.598	0.009
II	58	52		
III	42	34		
IV	15	6		
Anatomic location				
L-Lower	37	37	8.344	0.138
L-Upper	52	57		
R-Lower	41	46		
R-Middle	14	5		
R-Upper	76	86		
Uncertain	9	3		

Bidirectional disordered data were tested by pearson Chi-square test. Unidirectional ordered data (T, N, TNM, G) were tested by the rank-sum test of the sequential list (Mann–Whitney U test).

*TNM* tumor node metastasis.

### MiR-1976 targets NCAPH 3’-UTR: 1627 bp-1633 bp

**Figure 5:**
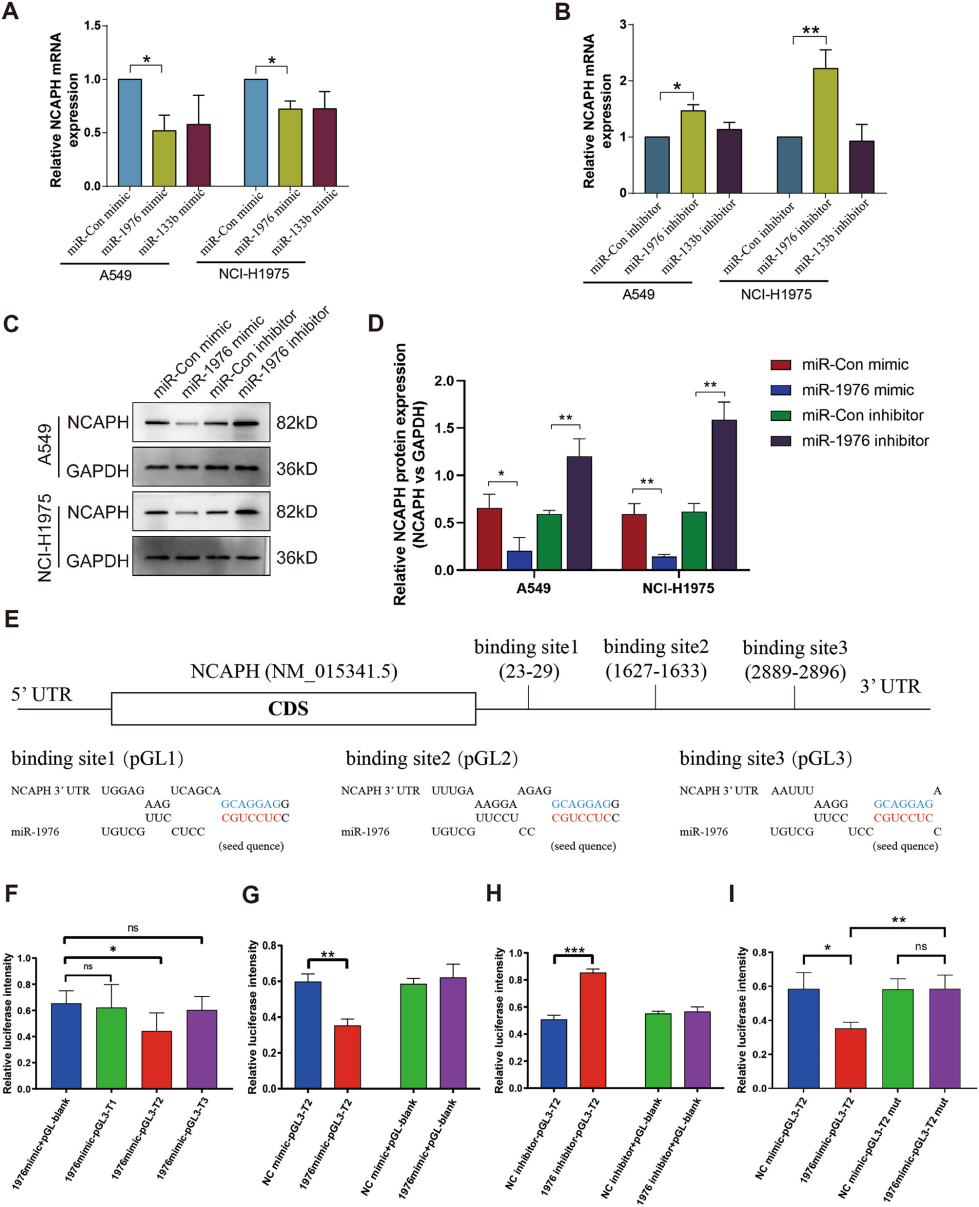
MiR-1976 targets NCAPH 3’-UTR: 1627 bp-1633 bp. A549 and NCI-H1975 cells were transfected with corresponding mimics, respectively and real-time PCR was used to detect the NCAPH mRNA level (**A**). A549 and NCI-H1975 cells were transfected with corresponding inhibitors, respectively and real-time PCR was used to detect the NCAPH mRNA level (**B**). Western blot was used to analyze NCAPH protein levels in A549 and NCI-H1975 cells (**C**, **D**). A schematic diagram showed the three predicted potential binding sites of miR-1976 at the 3 ' -UTR of NCAPH (**E**). The luciferase reporter vector containing predicted three binding sites were cloned into pGL3-promoter, namely pGL3-T1, pGL3-T2, and pGL3-T3. The three vectors were co-transfected with miR-1976 mimic respectively into A549 cells, and luciferase signals were detected by luciferase assay (**F**). The pGL3-T2 was co-transfected with the miR-1976 mimic into A549 cells. Luciferase signals were detected by luciferase assay (**G**). The pGL3-T2 was co-transfected respectively with the miR-1976 inhibitor into A549 cells. Luciferase signals were detected by luciferase assay (**H**). The pGL3-T2 was co-transfected with the miR-1976 mimic into A549 cells. The pGL3-T2 mut was co-transfected with the miR-1976 mimic into A549 cells. Luciferase signal was detected by luciferase method (**I**). * *P* < 0.05, ** *P* < 0.01, ***P < 0.001.

To test the ability of miR-1976 and miR-133b to regulate NCAPH expression in human LUAD cell lines, overexpression/knockdown of the corresponding miRNAs was performed by transducing miR-1976 mimic/inhibitor, miR-133b mimic/inhibitor into LUAD cells. After transfection with the miR-1976 mimic relative to the mimic control, NCAPH mRNA levels were markedly decreased (Fig. 5A, P < 0.05). NCAPH mRNA levels, however, were considerably more significant in the group treated with the miR-1976 inhibitor than in the control group (Fig. 5B, P < 0.001). After miR-1976 mimic/inhibitor transfection, NCAPH protein levels were correlated with mRNA levels (Fig. 5C,D, P < 0.001). However, miR-133b mimic/ miR-inhibitor overexpression did not change the NCAPH mRNA levels (Fig. 5A,B). The above data indicated that miR-1976, but not miR-133b, targets NCAPH expression.

Next, to detect whether miR-1976 directly binds to NCAPH 3 ' -UTR, we predicted the binding sites of miR-1976 in Targetscan, and a luciferase signal was detected by luciferase assay. (Fig. 5E). Compared to the pGL-blank group, co-transfection of miR-1976 mimic and pGL3-T2 significantly reduced the luciferase activity, and co-transfection of miR-1976 mimic and pGL3-T1 or pGL3-T3 did not change luciferase activity (Fig. 5F, P < 0.05). When the miR-1976 mimic and pGL3-T2 were co-transfected, we discovered that it dramatically decreased luciferase activity compared to the control mimic (Fig, 5G, P < 0.01). In contrast, the reporter vector pGL3-T2's luciferase activity was markedly elevated by miR-1976 suppression (Fig. 5H, P < 0.001). However, when we modified pGL3-T2 to pGL3-T2 mut, miR-1976 did not influence luciferase activity (Fig. 5I, P < 0.01). To summarize, miR-1976 inhibits NCAPH expression by directly binding to the 1627 bp-1633 bp of the NCAPH 3'UTR.

### MiR-1976 inhibits LUAD cell proliferation & migration and promotes cell apoptosis by targeting NCAPH in vitro

**Figure 6:**
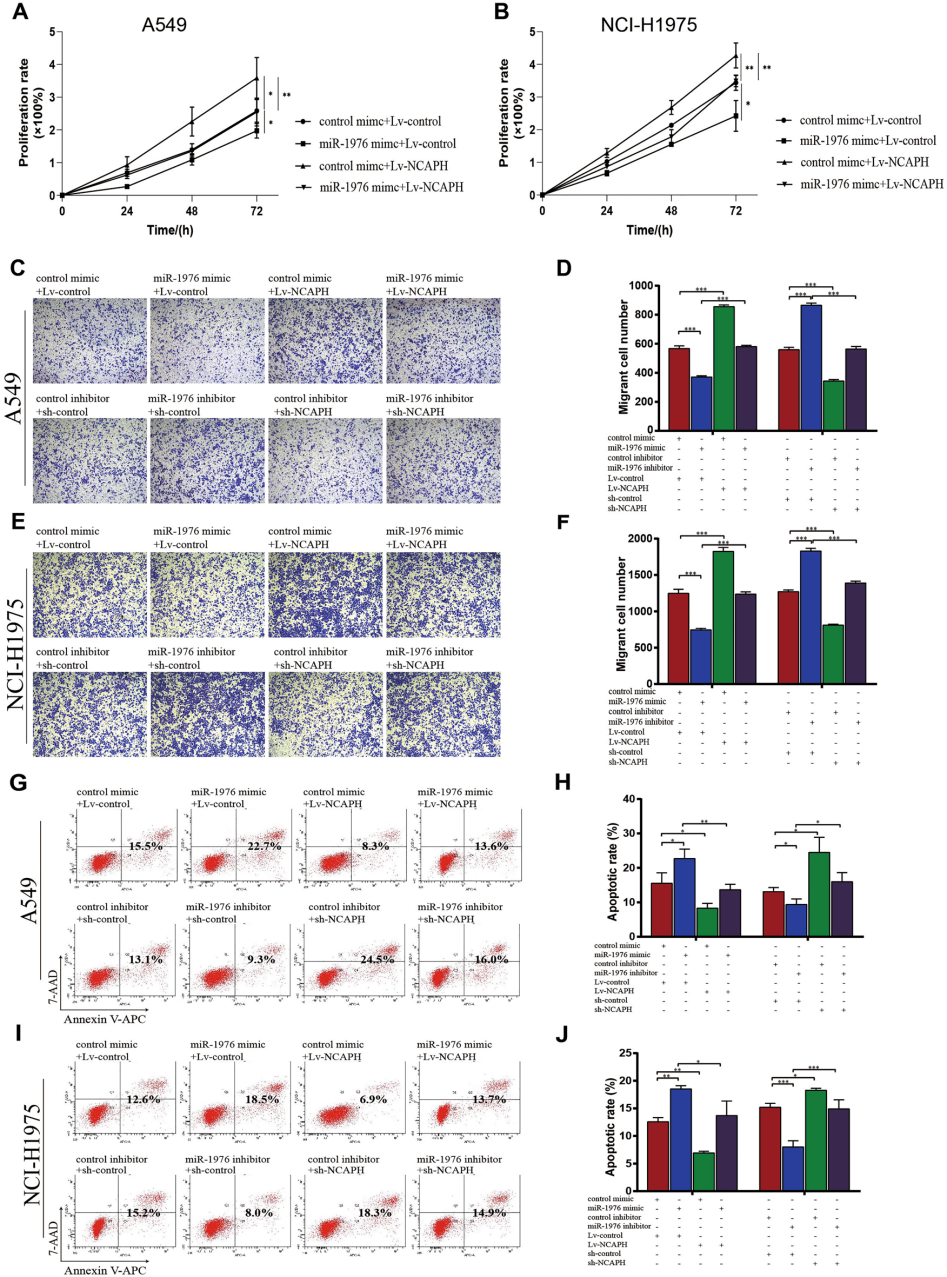
MiR-1976 inhibits LUAD cell proliferation & migration and promotes cell apoptosis by targeting NCAPH in vitro. The A549 or NCI-H1975 cells were transfected with NCAPH knockdown/overexpression lentivirus or miR-1976 mimic/inhibitor, respectively. The viability of LUAD cells was detected by CCK8 assay (**A**, **B**), the migration was performed by Transwell assays (**C**–**F**), and the apoptosis was measured by flow cytometry (**G**–**J**). Independent repeat n = 3. Data expressed as mean ± SEM. * *P* < 0.05, ** *P* < 0.01, ***P < 0.001.

Next, we investigated how miR-1976 behaved biologically in lung adenocarcinoma cells A549 and NCI-H1975 proliferation & migration. NCAPH expression significantly promoted A549 and NCI-H1975 cell proliferation compared to the control, whereas miR-1976 inhibited cell proliferation. Transfection of miR-1976 reversed the effect of NCAPH-induced cell proliferation (Fig. 6A,B, P < 0.01, P < 0.05). Compared to the control, miR-1976 mimic-transfected A549 cells showed less migration, and NCAPH overexpression promoted A549 cell movement. The migration-promoting ability of A549 cells induced by NCAPAH was reversed after miR-1976 transfection (Fig. 6C upper panel, 6D left histogram, P < 0.001). However, compared to control transfected cells, miR-1976 inhibitor transfected cells moved more, and the knockdown of NCAPH could prevent cell migration (Fig. 6C lower panel, 6D right histogram, P < 0.001). In contrast to A549 cells transfected with either miR-1976 inhibitor or sh-NCAPH alone, co-transfected A549 cells with miR-1976 inhibitor and sh-NCAPH they have regained migratory cell ability (Fig. 6C lower panel, 6D right histogram, P < 0.001). The findings show that miR-1976 specifically targeted NCAPH to impede LUAD cell migration. Similar results were confirmed in the NCI-H1975 cell (Fig. 6E,F, P < 0.001).

The level of cell apoptosis was then determined using apoptosis studies. Compared to the control mimic, the miR-1976 mimic boosted the apoptotic effect of A549 cells (Fig. 6G upper panel, 6H left histogram, P < 0.05). Contrary to the control lentivirus group, however, overexpression of the Lv-NCAPH lentivirus prevented cell apoptosis (Fig. 6G upper panel, 6H left histogram, P < 0.05). Following transfection with Lv-NCAPH lentivirus, pro-apoptotic effects brought on by the miR-1976 mimic were reversed in A549 cells (Fig. 6G upper panel, 6H left histogram, P < 0.05). The miR-1976 inhibitor decreased apoptosis, whereas the sh-NCAPH lentivirus increased apoptosis compared to the comparable control groups (Fig. 6G lower panel, 6H right histogram, P < 0.05). However, sh-NCAPH and the miR-1976 inhibitor co-transfected cells restored cell apoptosis (Fig. 6G lower panel, 6H right histogram, P < 0.05). Similar outcomes were seen when the procedures were repeated in NCI-H1975 cells (6I, 6 J, P < 0.05). In summary, miR-1976 controls LUAD cell proliferation, migration, and apoptosis by inhibiting NCAPH.

### The miR-1976 inhibits tumor growth in vivo by targeting NCAPH

**Figure 7:**
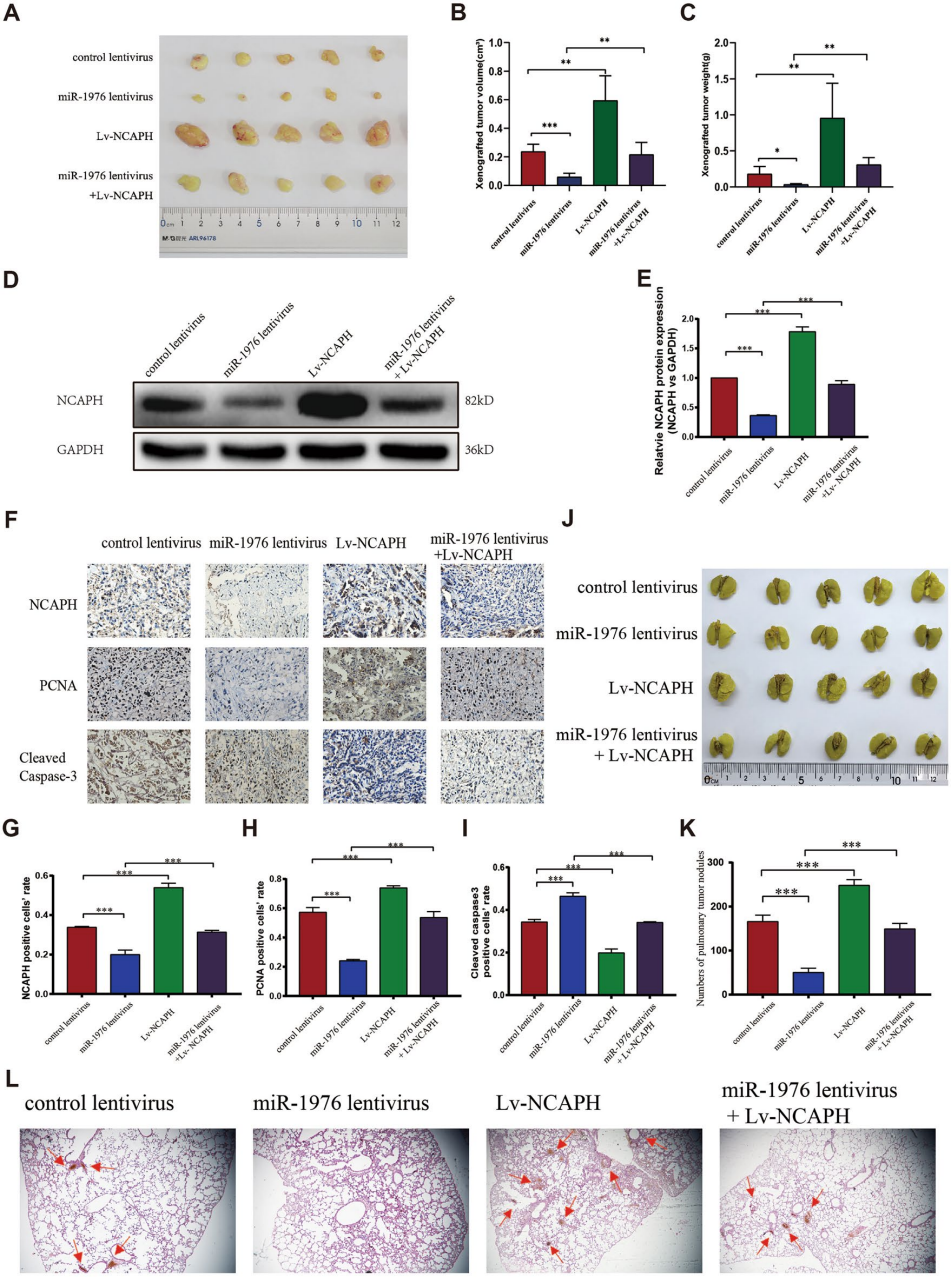
MiR-1976 suppresses tumor growth & metastasis in vivo by targeting NCAPH. A549 cells were infected with the corresponding lentivirus, respectively. About 1 × 10^7^ cells were implanted subcutaneously into nude mice (**A**), and the tumor volume (**B**) and weight(**C**) were observed after cell implantation. NCAPH protein level in xenograft tumor tissues was detected by Western blotting (**D**, **E**). In Fig. 7D, the experiments were repeated twice on one membrane (Supplementary information file 2), and Fig. 7D represented one experimental result. The expression of NCAPH, PCNA, and cleaved-caspase-3 in xenograft tumors was detected by IHC staining (**F**–**I**). PCNA is a marker of tumor proliferation. A549 cells infected with lentivirus were injected into the tail vein of nude mice (5 in each group) (**J**). The average number of tumor nodules in each group was calculated (K). HE staining was used to demonstrate tumor nodules (L). * *P* < 0.05, ** *P* < 0.01, ***P < 0.001.

To verify the above data in vivo, we further established the Xenograft model by subcutaneously implanting A549 cells into BALB/c immune-deficient mice for four weeks. We discovered that the tumor volume of the miR-1976 lentivirus group decreased compared to the control lentivirus group. In contrast, the tumor volume of the Lv-NCAPH lentivirus group considerably increased (Fig. 7A,B, P < 0.01). However, the tumor volume in the miR-1976 + Lv-NCAPH lentivirus group regained the control level. (Fig. 7A,B, P < 0.05). The tumor weight had similar findings (Fig. 7C, P < 0.05). These findings suggested that overexpressing miR-1976 could reduce the growth-promoting effects of NCAPH in vivo.

Then the total proteins of the tumor tissues in nude mice were extracted, and Western blot assays were used to find NCAPH protein expressions in all of the groups mentioned above. Compared to the control group, a significant down-regulated NCAPH protein level was observed in the miR-1976 high-expression group. NCAPH level was significantly up-regulated in the Lv-NCAPH lentivirus group compared to the control group (Fig. 7D,E, P < 0.001). The re-introduction of Lv-NCAPH lentivirus partially reversed the negative-regulation effect of miR-1976 on NCAPH expression (Fig. 7D,E, P < 0.001). Changes in NCAPH and cell proliferative and apoptotic indicators were also discovered using immunochemistry labeling in tumor samples. (Fig. 7F). As shown in Fig. 6F–G, NCAPH expression was lower in the miR-1976 over-expression group compared to the control lentivirus group. In contrast, NCAPH-positive cells were increased in the Lv-NCAPH lentivirus group (Fig. 7G, P < 0.001). Simultaneously, the PCNA expression showed the same trends as NCAPH protein expression (Fig. 7H, P < 0.001), while cleaved-caspase-3 displayed the opposite trends to NCAPH protein expression (Fig. 7I, P < 0.001). Notably, Lv-NCAPH + miR-1976 lentivirus co-transfection restored the expression of NCAPH, PCNA, and cleaved-caspase-3 to the control levels (Fig. 7G,H,I, P < 0.001).

### The mir-1976 inhibits tumor metastasis by targeting NCAPH in vivo

To further comprehend how miR-1976 affected LUAD metastasis, we injected A549 cells into the tail vein to generate a lung cancer metastasis model. The findings revealed that the number of tumor nodules was significantly lower in the miR-1976 lentivirus group than in the control group. The number of nodules considerably increased in the NCAPH lentivirus group compared to the control (Fig. 7J,K, P < 0.001). The effect of the mir-1976-induced decrease in the number of tumor nodules was considerably reversed by the overexpression of NCAPH (Fig. 7J,K, P < 0.001). The HE staining experiment produced identical results (Fig. 7L). The studies above revealed that miR-1976 reduced LUAD metastases by in vivo targeting NCAPH expression.

### MiR-1976 targeting NCAPH blocks the activation of NF-kB

**Figure 8:**
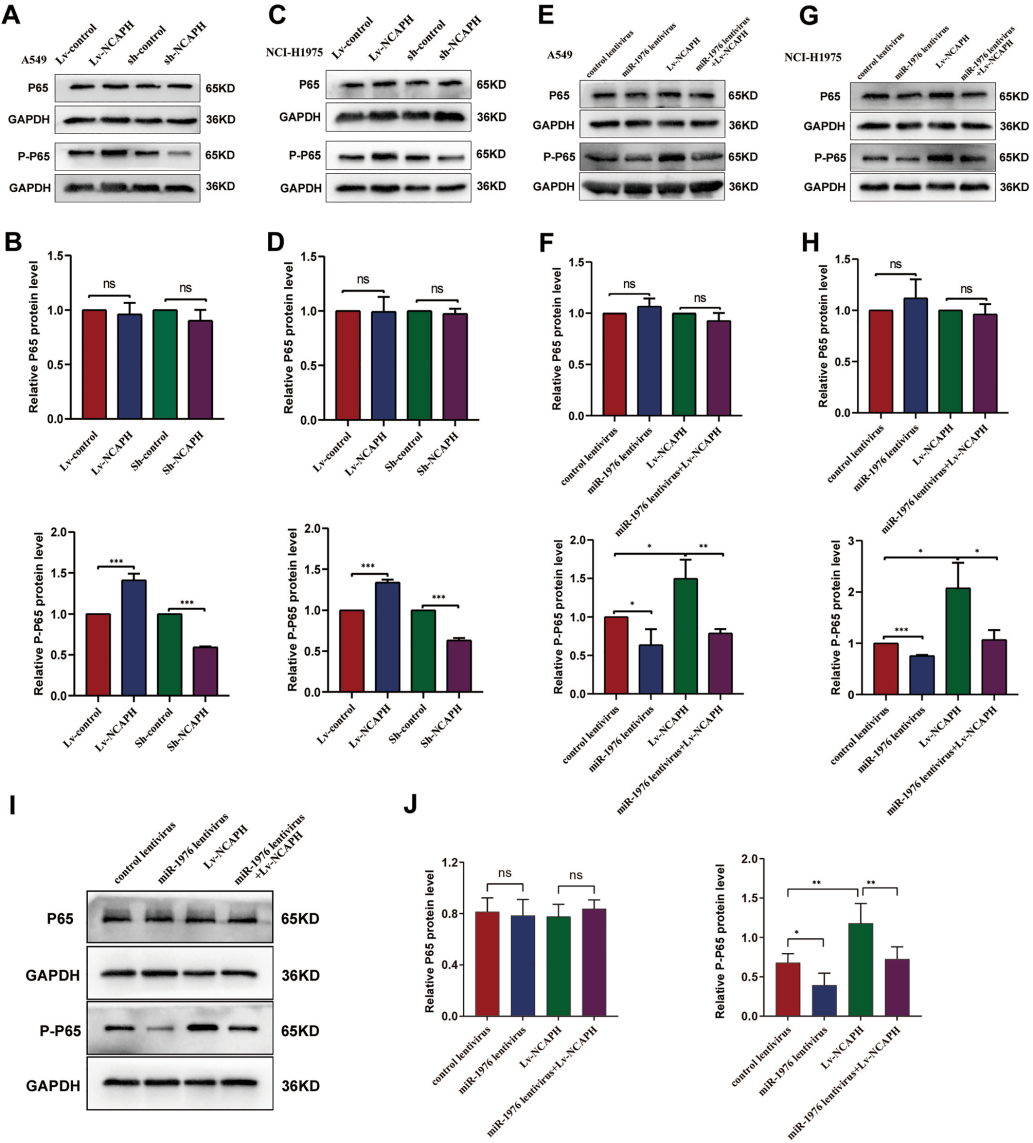
MiR-1976 targeting NCAPH blocks the activation of NF-kB. The A549 cells and NCI-H1975 cells were infected with the corresponding lentivirus, respectively. The protein levels of p65 and phosphorylated-p65 were detected by western blot assay and quantitative analysis in A549 cells (**A**, **B**, **E**, **F**) and NCI-1975 cells (**C**, **D**, **G**, **H**). About 1 × 10^7^ cells infected with the corresponding lentivirus, were implanted subcutaneously into nude mice. The protein levels of p65 and phosphorylated-p65 were detected by western blot assay and quantitative analysis in each xenograft group (I, J). * P < 0.05, **P < 0.01, ***P < 0.001.

**Figure 9:**
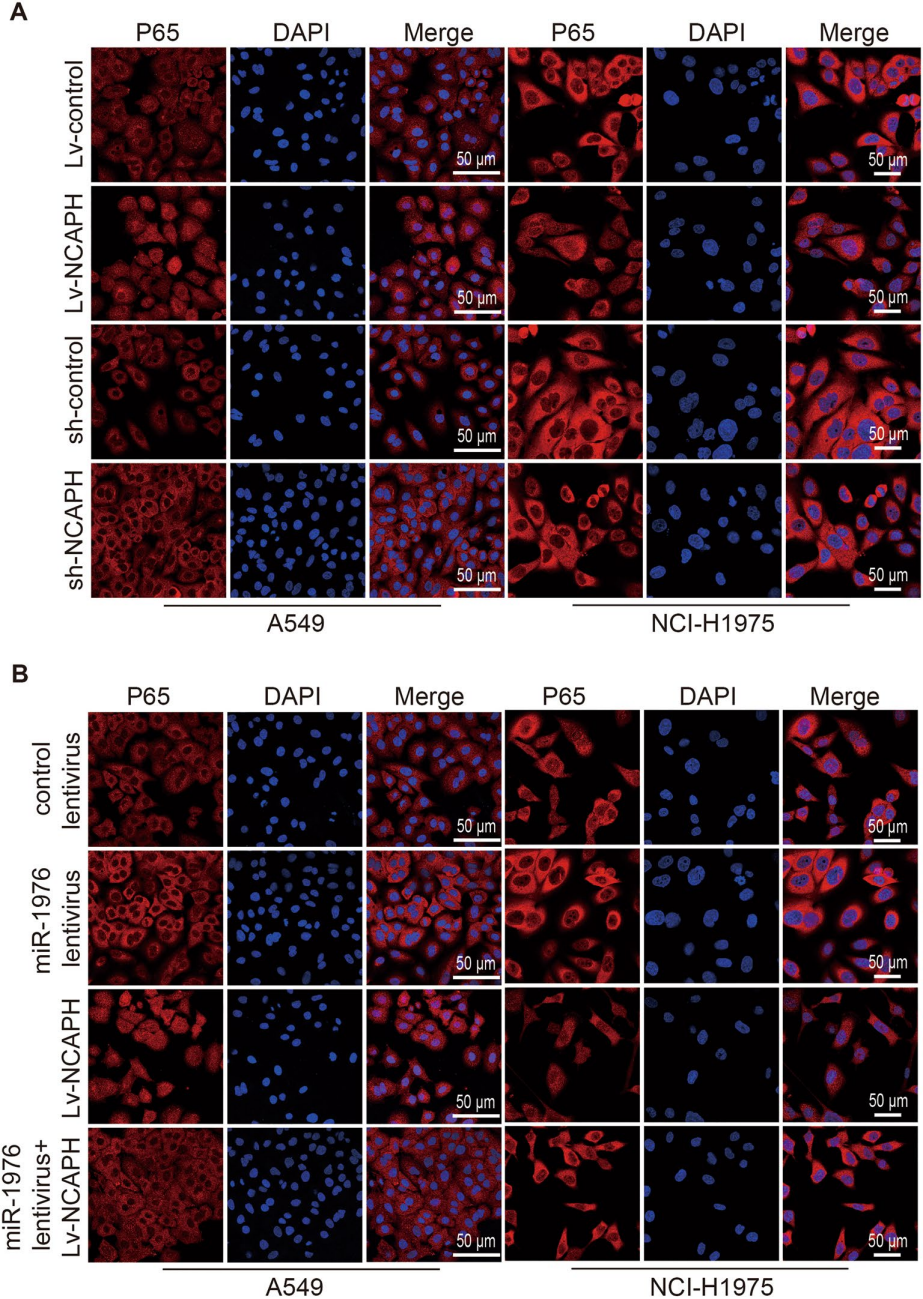
MiR-1976 targeting NCAPH blocks the activation of NF-kB. The A549 cells and NCI-H1975 cells were infected with the corresponding lentivirus, respectively. The levels of p65 in cytoplasm and nucleus were detected by Laser confocal microscope (A, B). DAPI is used to stain the nucleus. The scale is 50 nm.

Studies have revealed that the NF-kB pathway involves cell proliferation, migration, and other biological processes. To further explore the downstream pathway induced by the miR-1976/NCAPH axis, we detected the activation of NF-kB. The expression of phosphorylated p65 was shown to be up-regulated by NCAPH overexpression and down-regulated by NCAPH inhibition (Fig. 8A,B, P < 0.001). Despite NCAPH overexpression and knockdown, the overall level of p65 protein remained the same (Fig. 8A,B, P > 0.05, P < 0.001). The above results were confirmed in the NCI-1975 cell line (Fig. 8C,D, P < 0.001). In addition, laser confocal analysis of LUAD cells showed that P65 significantly entered the nucleus from cytoplasm after overexpression of NCAPH (Fig. 9A). Notably, compared to the control, the overexpression of miR-1976 reduced the phosphorylation of p65. Meanwhile, the overexpression of miR-1976 could counteract an increase in phosphor-p65 level caused by the overexpression of NCAPH. (Fig. 8E–H, P < 0.05, P < 0.01, P < 0.001). Similarly, laser confocal showed that overexpression of miR-1976 significantly reduced NCAPH-induced P65 entry into the nucleus from cytoplasm (Fig. 9B).

## Discussion

Lung cancer is one of the most aggressive malignancies threatening human life and health^24^. As the population has become older, environmental pollution has gotten worse, and more people are smoking, the prevalence of lung cancer has been rising annually^25^. Surgery is the primary treatment option for early-stage lung cancer^26^. The onset of lung adenocarcinoma is insidious, and the early symptoms are atypical, so many patients miss the best treatment opportunity when they are diagnosed^27^. Recently, significant advances have been made in the molecular genetics and immunotherapy of lung adenocarcinoma, which brings new promise to patients suffering from lung adenocarcinoma^28^. Till now, NCAPH, as an oncogene, has been implicated in multiple dangerous cancer, such as breast cancer^7^, endometrial cancer^8^, and NSCLC^9^. In this study, we found that the expression of NCAPH was up-regulated in lung adenocarcinoma compared to standard control based on the TCGA and GEO database (Fig. 1A–C).

Meanwhile, 14 LUAD specimens had significantly greater NCAPH mRNA levels and protein expression than comparable neighboring tissues (Fig. 1D,E,F). Moreover, lung cancer patients' survival was strongly correlated with the expression of NCAPH (Fig. 1G). According to recent investigations, NCAPH appears to be involved in various tumor cell biological activities. In pancreatic cancer (PC) cell lines, the down-regulated NCAPH inhibited PC cell proliferation and colony formation. NCAPH also plays a vital role in cell cycle progression and DNA damage. NCAPH downregulation induced cell apoptosis via a caspase-dependent Pathway^29^. NSCLC cells' invasion, migration, and colony formation were decreased by NCAPH knockdown, which also prevented their proliferative growth and caused a G2/M cell cycle stop. NCAPH was associated with the development and progression of lung adenocarcinoma and represented a potential therapeutic target in lung adenocarcinoma^29,30^. Our current research showed that overexpressing NCAPH greatly encouraged proliferation and migration and prevented apoptosis in LUAD cells (Fig. 2E–L). These findings revealed that NCAPH plays an oncogenic role in LUAD. In addition, we also found that NCAPH can promote DNA replication activity and cell enrichment in the S phase of LUAD cells (Fig. 3). In the study of Li et al. it was also found that inhibiting the expression of NCAPH could reduce the proliferation of lung adenocarcinoma cells, which is consistent with our findings^31^. However, how NCAPH is involved in the progression of lung adenocarcinoma remains unclear.

Our following question is why and how NCAPH is increased in LUAD tissues. Several studies have found that the miRNA-mRNA regulatory network is linked to carcinogenesis and survival prognosis in various cancers. Because the levels of miRNAs differ significantly between normal and malignant tissues, many of them can be employed as biomarkers for prognosis and prediction^32^. We hypothesized that microRNAs could alter NCAPH post-transcriptionally. Several miRNAs are involved in complicated regulatory networks. Many miRNAs can control a single gene, and a single miRNA can target multiple candidates^33^. Previous research found that miRNA-133b inhibited osteosarcoma cell motility, migration, invasion, and EMT by targeting FGFR1^34^. In NSCLC, miR-133b targeting NCAPH boosted β-catenin degradation while decreasing cancer stem cell maintenance^35^. Gang Chen et al. discovered that miR-1976 functioned as a tumor suppressor in the evolution of NSCLC and directly targeted PLCE1^23^. The TargetScan and linkedomics datasets were combined in our investigation to identify the potential microRNAs miR-1976 and miR-133b that target NCAPH (Fig. 4A). Real-time PCR revealed that miR-1976 and miR-133b levels in LUAD tissues were lower than in nearby normal tissues (Fig. 4B,C). Furthermore, Pearson's correlation test revealed a strong negative association between NCAPH mRNA levels and miR-1976 expression (Fig. 4D,R=  − 0.8154, P < 0.001). However, miR-133b level was positively correlated with NCAPH mRNA level (Fig. 4E, R = 0.8758, P < 0.001). The TCGA data also confirmed the negative correlation of miR-1976 level with NCAPH mRNA level (Fig. 4G, R =  − 0.228, P < 0.001). In addition, we used the TCGA dataset to explore the prognostic value of miR-1976. The result showed that the prognosis of patients with high expression of miR-1976 (n = 229) was significantly better than that of patients with low expression of miR-1976 (n = 231) (Fig. 4H).

Then, we further found that miR-1976 inhibited the expression of NCAPH. However, the NCAPH mRNA level could not be regulated by miR-133b (Fig. 5A,B). We designed luciferase reporter assays to explore further which part of NCAPH is targeted by miR-1976 to impair the NCAPH expression. We first predicted three binding sites of NCAPH 3 ′UTR region, which might be targeted by miR-1976 using the Targetscan database. We named them binding site1, binding site2, and binding site 3, respectively (Fig. 5E). We found only binding site 2 (1627 bp-1633 bp sites of NCAPH 3’-UTR) was targeted by miR-1976(Fig. 5F–H), that is miR-1976 downregulated NCAPH expression by binding to the 1627 bp-1633 bp of NCAPH 3’UTR. Next, by constructing a mutant of binding site 2 and co-transfecting lung adenocarcinoma cells with miR-1976, it was found that miR-1976 could not inhibit luciferase activity, thus further proving the binding of miR-1976 to NCAPH binding site 2. Then we found that miR-1976 did suppress the proliferation and migration and promoted apoptosis of LUAD cells via targeting NCAPH in vivo (Fig. 6). Following that, we discovered that the mir-1976/NCAPH axis plays a critical role in tumor growth and metastasis (Fig. 7) in mouse models. Xiong et al. have shown that miR-133b targeted NCAPH and performed a critical role in the progression of NSCLC^35^. However, we found that miR-1976, but not miR-133b, targeting NCAPH, was involved in lung adenocarcinoma's growth, metastasis, and apoptosis.

It is understood that NF-κB is a crucial participant in many stages of cancer development and progression^36,37^. We found that the overexpression of miR-1976 reduced the phosphorylation of p65, one of the NF-κB activation signals. Meanwhile, the overexpression of miR-1976 could counteract an increase in phosphor-p65 level caused by the overexpression of NCAPH (Figs. 8, 9). According to the findings, miR-1976 selectively targeted NCAPH and then inhibited NF-κB activation. We attempt to explore how NCAPH promoted the phosphorylation of p65. We assumed that NCAPH might bind to IκB, an NF-κB inhibitor, decreasing its inhibitory activity and ultimately increasing p65 phosphorylation. Unfortunately, the Co-IP results revealed that NCAPH could not bind to IκB (supplementary Fig. 1A, B). In addition, NCAPH was not found to interact with P65 (supplementary Fig. 1C, D). The CoIP-MS (mass spectrometry) analysis will be undertaken to discover how NCAPH stimulates p65 phosphorylation.

## Conclusion

**Figure 10:**
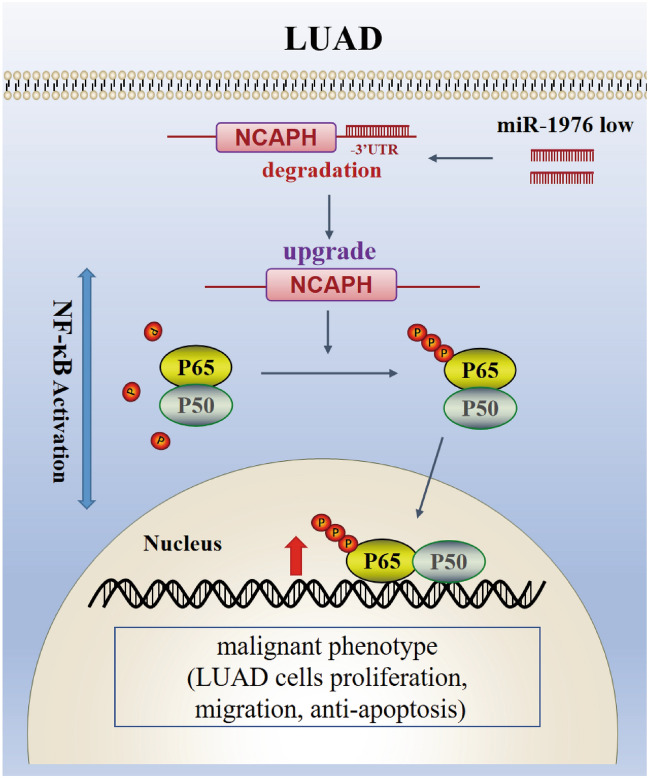
A diagram shows that MiR-1976 suppresses LUAD via NCAPH/ NF-κB pathway. MiR-1976, which can bind to the NCAPH-3 'UTR terminal, is reduced in LUAD cells, resulting in increased NCAPH mRNA and protein expression in lung cancer. NCAPH overexpression stimulates p65 phosphorylation, which activates the NF-pathway and promotes the development of malignant features in LUAD cells (proliferation, migration, and anti-apoptosis).

In conclusion, this study demonstrates that miR-1976, targeting NCAPH, can operate as a tumor suppressor and decrease the malignant phenotypes of lung adenocarcinoma. The miR-1976 binding site that controls NCAPH is found at 1627 bp-1633 bp of the NCAPH 3'-UTR (Fig. 10). The miR-1976/NCAPH/NF-B axis could represent helpful future diagnostic and prognostic biomarkers and intriguing treatment prospects.

### Supplementary Information

Supplementary Information.

## Data Availability

The bioinformatics datasets generated and/or analysed during the current study are available in the TCGA (https://cancergenome.nih.gov) and GEO (https://www.ncbi.nlm.nih.gov/geo/) databases. The experimental data sets used and analyzed in this study are available from the corresponding authors.

## Abbreviations

LUAD: Lung adenocarcinoma
OS: Overall survival
UTR: Untranslated regions
NSCLC: Non-small cell lung cancer
SCLC: Small cell lung cancer
NCAPH: Non-SMC Condensin I complex subunit H
MiR: MicroRNA
EMT: Epithelial-mesenchymal transition
HRP: Horseradish peroxidase; H&E, Hematoxylin–Eosin Staining
IHC: Immunohistochemical Staining
PC: Pancreatic cancer

## References

[1] Angrisani A, Di Fiore A, De Smaele E, Moretti M (2021). The emerging role of the KCTD proteins in cancer. Cell. Commun. Signal.

[2] Torre LA, Siegel RL, Jemal A (2016). Lung cancer statistics. Adv. Exp. Med. Biol..

[3] Miller KD (2019). Cancer treatment and survivorship statistics, 2019. CA Cancer J. Clin..

[4] Siegel RL, Miller KD, Fuchs HE, Jemal A (2021). Cancer Statistics, 2021. CA Cancer J. Clin..

[5] Cabello OA, Baldini A, Bhat M, Bellen H, Belmont JW (1997). Localization of BRRN1, the human homologue of Drosophila barr, to 2q11.2. Genomics.

[6] Viera A (2007). Condensin I reveals new insights on mouse meiotic chromosome structure and dynamics. PLoS One.

[7] Lu H (2020). Identification of NCAPH as a biomarker for prognosis of breast cancer. Mol. Biol. Rep..

[8] Qiu X, Gao Z, Shao J, Li H (2020). NCAPH is upregulated in endometrial cancer and associated with poor clinicopathologic characteristics. Annal. Hum. Genet..

[9] Ma Q (2019). Identification and validation of key genes associated with non-small-cell lung cancer. J. Cell. Physiol..

[10] Yin L (2017). NCAPH plays important roles in human colon cancer. Cell. Death Dis..

[11] Cui F, Hu J, Xu Z, Tan J, Tang H (2019). Overexpression of NCAPH is upregulated and predicts a poor prognosis in prostate cancer. Oncol. Lett..

[12] Sun C (2019). Non-SMC condensin I complex subunit H enhances proliferation, migration, and invasion of hepatocellular carcinoma. Mol. Carcinog..

[13] Klinge CM (2015). miRNAs regulated by estrogens, tamoxifen, and endocrine disruptors and their downstream gene targets. Mol Cell. Endocrinol..

[14] Zhou RS (2019). Integrated analysis of lncRNA-miRNA-mRNA ceRNA network in squamous cell carcinoma of tongue. BMC Cancer.

[15] Tutar Y (2014). miRNA and cancer; computational and experimental approaches. Curr. Pharm. Biotechnol..

[16] Zhang L, Liao Y, Tang L (2019). MicroRNA-34 family: A potential tumor suppressor and therapeutic candidate in cancer. J. Exp. Clin. Cancer Res..

[17] Kobayashi T (2019). Editorial Comment to Micro-ribonucleic acid expression signature of metastatic castration-resistant prostate cancer: Regulation of NCAPH by antitumor miR-199a/b-3p. Int. J. Urol..

[18] Chen P (2019). Expression levels and co-targets of miRNA-126-3p and miRNA-126-5p in lung adenocarcinoma tissues: Αn exploration with RT-qPCR, microarray and bioinformatic analyses. Oncol. Rep..

[19] Tian Z (2016). MicroRNA-133b inhibits hepatocellular carcinoma cell progression by targeting Sirt1. Exp. Cell. Res..

[20] Li C (2016). miR-133b inhibits glioma cell proliferation and invasion by targeting Sirt1. Oncotarget.

[21] Wang J (2020). MiR-1976 knockdown promotes epithelial-mesenchymal transition and cancer stem cell properties inducing triple-negative breast cancer metastasis. Cell. Death Dis..

[22] Sahu SS (2019). The role and therapeutic potential of miRNAs in colorectal liver metastasis. Sci. Rep..

[23] Chen G, Hu J, Huang Z, Yang L, Chen M (2016). MicroRNA-1976 functions as a tumor suppressor and serves as a prognostic indicator in non-small cell lung cancer by directly targeting PLCE1. Biochem. Biophys. Res. Commun..

[24] Pallis AG, Syrigos KN (2013). Lung cancer in never smokers: Disease characteristics and risk factors. Crit. Rev. Oncol. Hematol..

[25] Hoy H, Lynch T, Beck M (2019). Surgical treatment of lung cancer. Crit. Care Nurs. Clin. North Am..

[26] Zhao H, Zhang X, Guo L, Shi S, Lu C (2021). A robust seven-gene signature associated with tumor microenvironment to predict survival outcomes of patients with Stage III-IV lung adenocarcinoma. Front. Genet..

[27] Subramanian J, Govindan R (2008). Molecular genetics of lung cancer in people who have never smoked. Lancet Oncol..

[28] Kim JH, Youn Y, Kim KT, Jang G, Hwang JH (2019). Non-SMC condensin I complex subunit H mediates mature chromosome condensation and DNA damage in pancreatic cancer cells. Sci. Rep..

[29] Kim B, Kim SW, Lim JY, Park SJ (2020). NCAPH Is Required for proliferation, migration and invasion of non-small-cell lung cancer cells. Anticancer Res..

[30] Nelson KM, Weiss GJ (2008). MicroRNAs and cancer: Past, present, and potential future. Mol. Cancer Ther..

[31] Li C, Meng J, Zhang T (2022). NCAPH is a prognostic biomarker and associated with immune infiltrates in lung adenocarcinoma. Sci. Rep..

[32] Li M, Li J, Ding X, He M, Cheng SY (2010). microRNA and cancer. AAPS J..

[33] Haas U, Sczakiel G, Laufer SD (2012). MicroRNA-mediated regulation of gene expression is affected by disease-associated SNPs within the 3'-UTR via altered RNA structure. RNA Biol..

[34] Gao G, Tian Z, Zhu HY, Ouyang XY (2018). miRNA-133b targets FGFR1 and presents multiple tumor suppressor activities in osteosarcoma. Cancer Cell. Int..

[35] Xiong Q (2021). miR-133b targets NCAPH to promote β-catenin degradation and reduce cancer stem cell maintenance in non-small cell lung cancer. Signal Transduct. Target. Ther..

[36] Hoesel B, Schmid JA (2013). The complexity of NF-κB signaling in inflammation and cancer. Mol. Cancer.

[37] Lalle G, Twardowski J, Grinberg-Bleyer Y (2021). NF-κB in cancer immunity: Friend or foe?. Cells.

