# Supplementary material for: MiR-1976/NCAPH/P65 axis inhibits the malignant phenotypes of lung adenocarcinoma
Source: Sci Rep. 2024 May 16;14:11211. doi: 10.1038/s41598-024-61261-6 (PMC11099075; doi:10.1038/s41598-024-61261-6)
Supplement: Supplementary file 1 — Supplementary Information. [file 41598_2024_61261_MOESM1_ESM.pdf]

Supplementary information 1：Supplementary figure 1

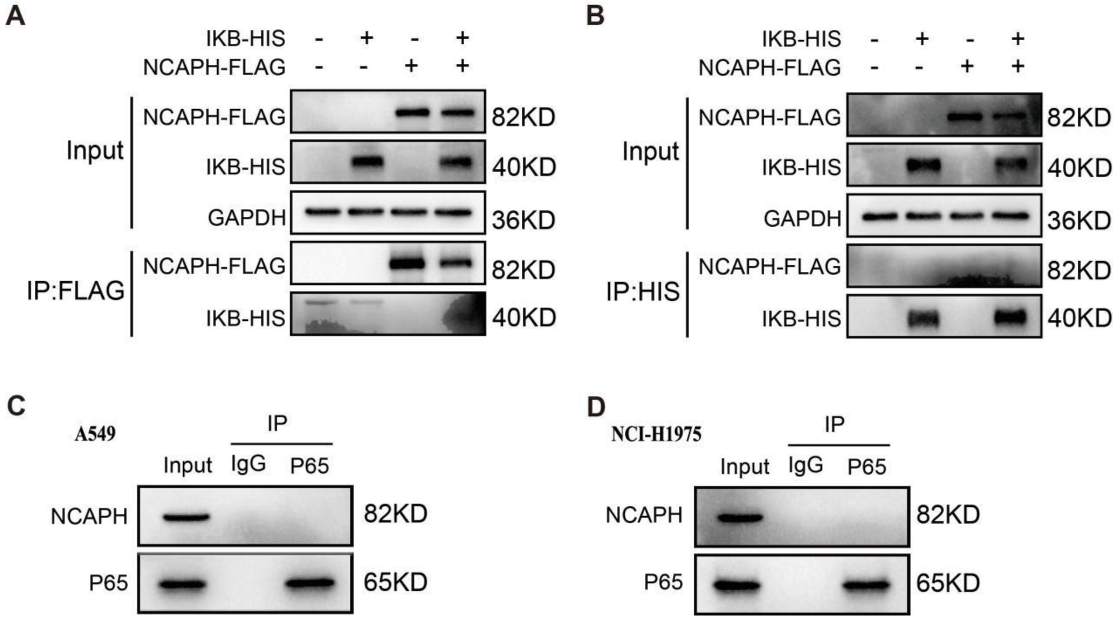

Supplementary figure 1

Supplementary figure 1: Immunoprecipitation analysis: Lung adenocarcinoma A549 cells were co-transfected with FLag-tagged NCAPH and His-labeled IKB. Magnetic beads bearing anti-FLAG (A) and anti-HIS (B) antibodies were used to separate the cell lysates. Endogenous immunoprecipitation analysis of NCAPH and P65 in A549 (C) and NCI-H1975 (D) cells. The SDS-PAGE analysis of the immunoprecipitates (IP group) and cell extracts (input group) was followed by immunoblotting using the designated antibodies.

Supplementary information 2： western blot results uncropped

1、Figure 2A

Repeat one：

NCAPH

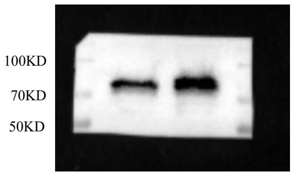

82KD

GAPDH

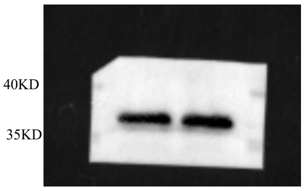

36KD

NCAPH

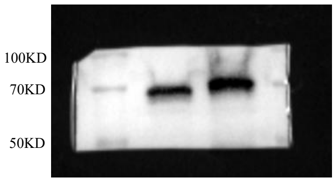

82KD

GAPDH

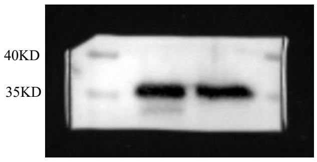

36KD

Repeat two：

NCAPH

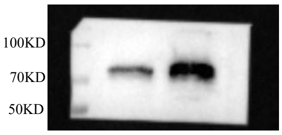

82KD

GAPDH

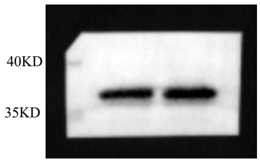

36KD

NCAPH

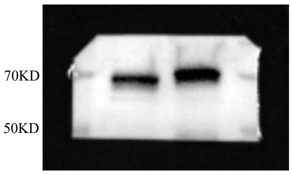

82KD

GAPDH

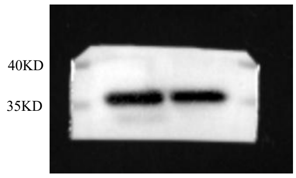

36KD

Repeat three：

NCAPH

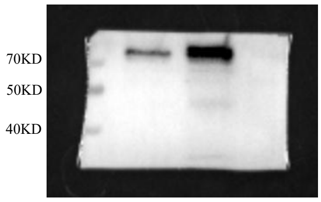

82KD

GAPDH

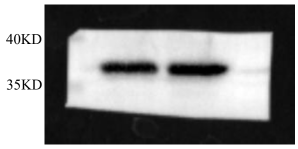

36KD

NCAPH

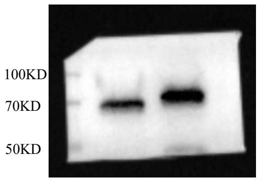

82KD

GAPDH

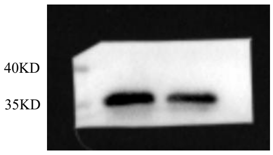

36KD

Repeat four：

NCAPH

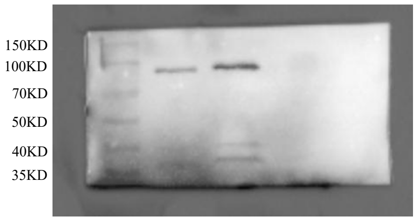

82KD

GAPDH 36KD

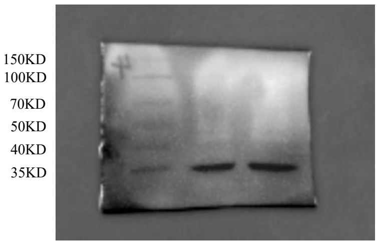

NCAPH 82KD

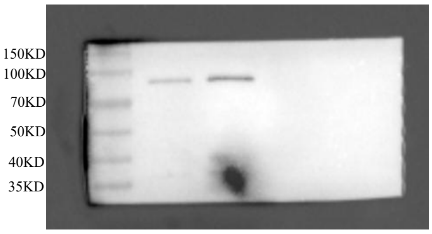

GAPDH 36KD

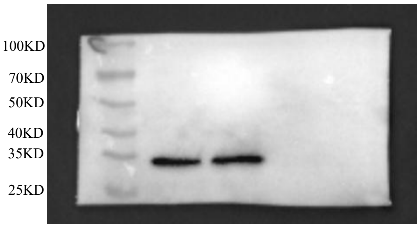

2、Figure 2C

Repeat one：

NCAPH 82KD

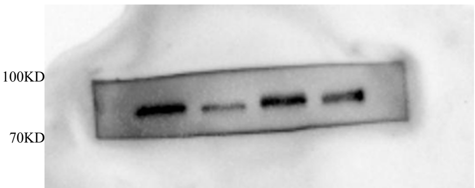

GAPDH 36KD

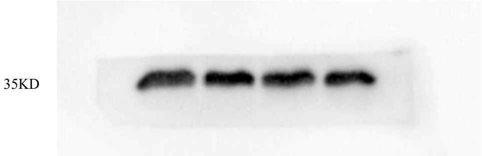

NCAPH 82KD

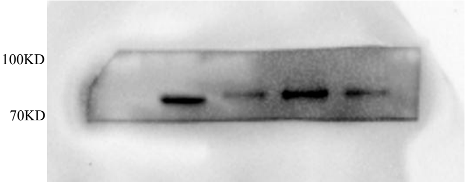

GAPDH 35KD 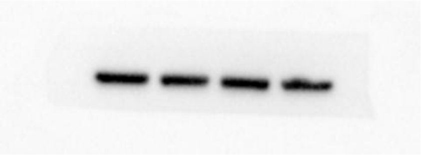 36KD

Repeat two：

NCAPH 70KD 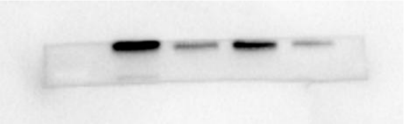 82KD

GAPDH 35KD 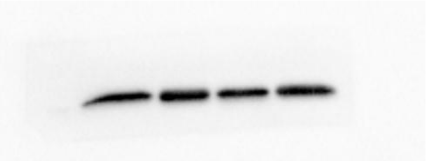 36KD

NCAPH 100KD 70KD 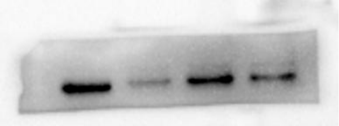 82KD

GAPDH 35KD 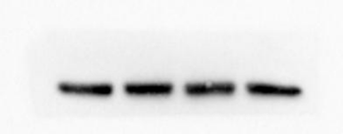 36KD

Repeat three：

NCAPH 100KD 70KD 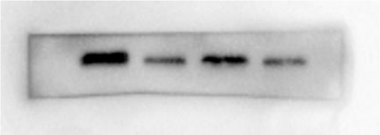 82KD

GAPDH 35KD 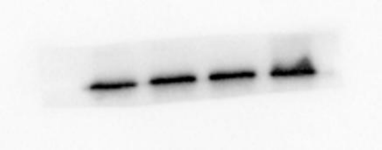 36KD

NCAPH 100KD 70KD 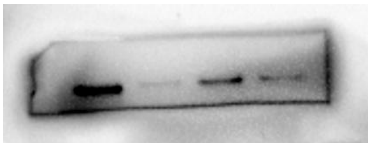 82KD

GAPDH 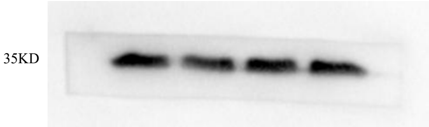

36KD

Repeat four：

NCAPH 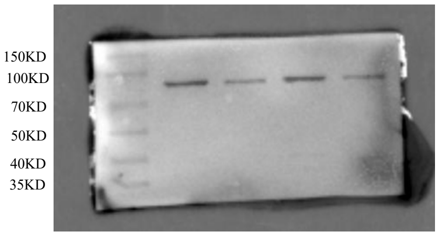

82KD

GAPDH 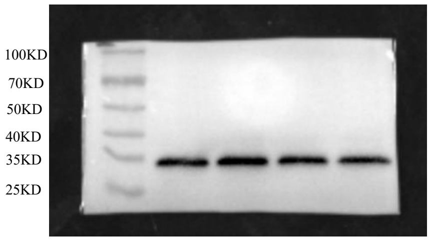

36KD

NCAPH 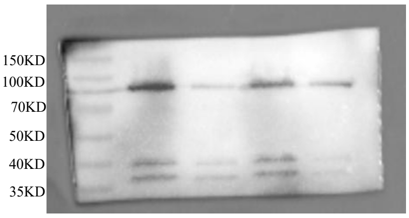

82KD

GAPDH 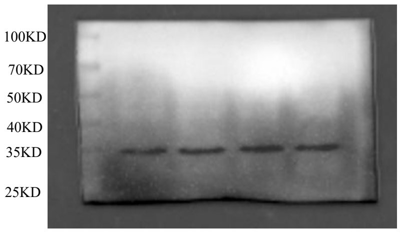

36KD

3、Figure 5C

Repeat one：

NCAPH

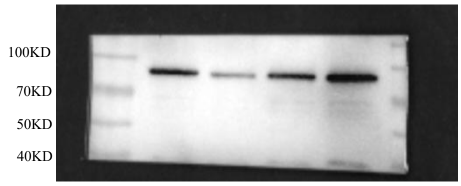

82KD

GAPDH

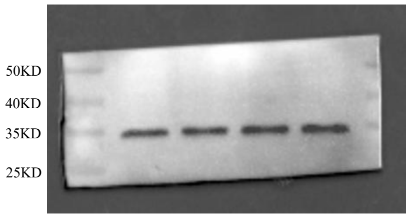

36KD

NCAPH

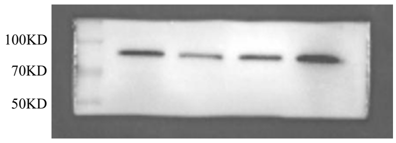

82KD

GAPDH

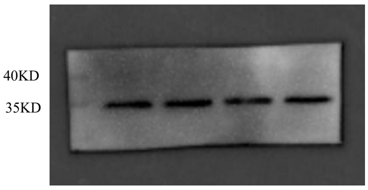

36KD

Repeat two：

NCAPH

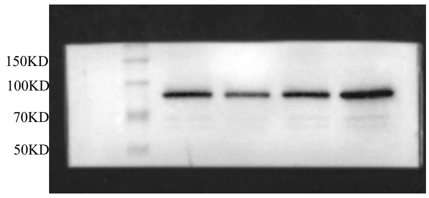

82KD

GAPDH

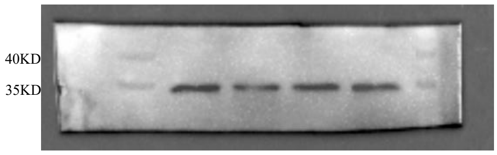

36KD

NCAPH

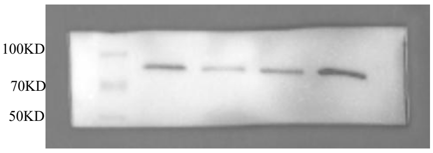

82KD

GAPDH

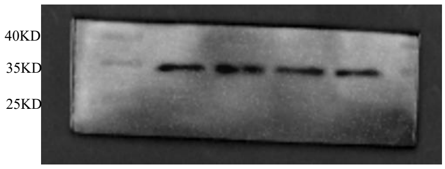

36KD

Repeat three：

NCAPH

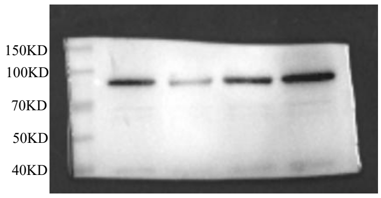

82KD

GAPDH

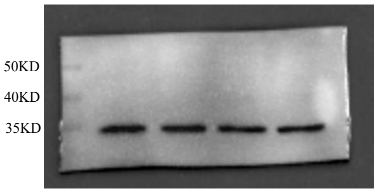

36KD

NCAPH

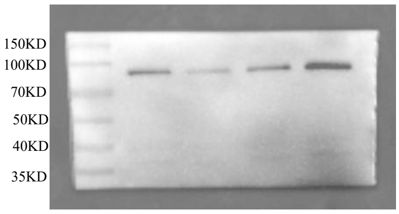

82KD

GAPDH

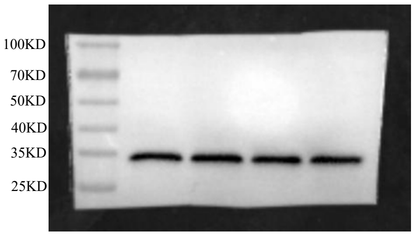

36KD

4、Figure 7D

Repeat one and two：

NCAPH

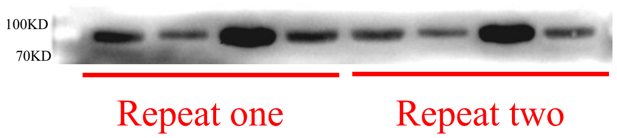

82KD

GAPDH

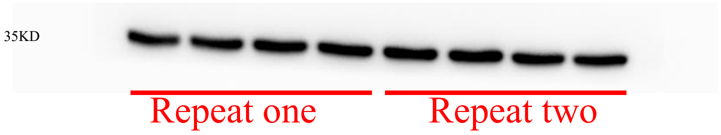

36KD

Repeat three and four：

NCAPH

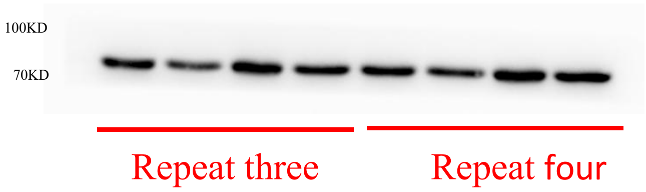

82KD

GAPDH

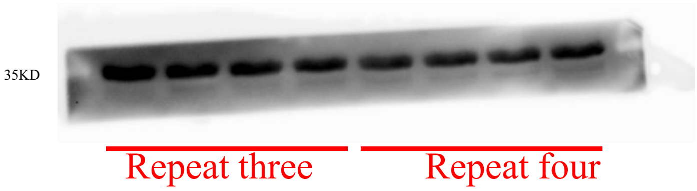

36KD

Repeat five：

NCAPH

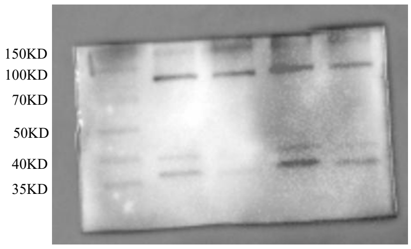

82KD

GAPDH

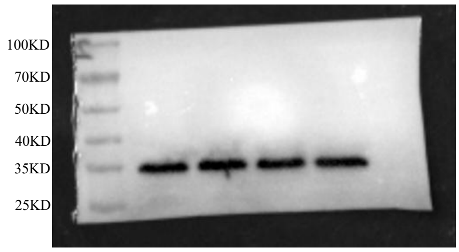

36KD

5、Figure 8A

Repeat one：

P65 70KD 50KD 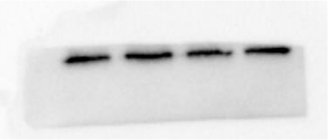 65KD

GAPDH 35KD 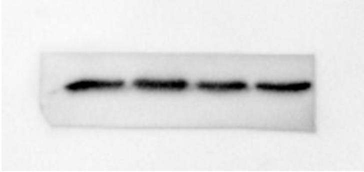 36KD

P-P65 70KD 50KD 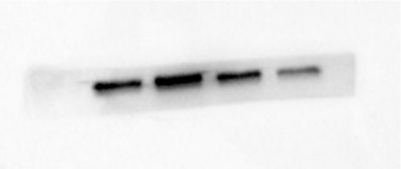 65KD

GAPDH 35KD 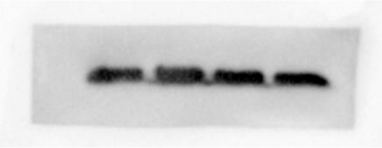 36KD

Repeat two：

P65 70KD 50KD 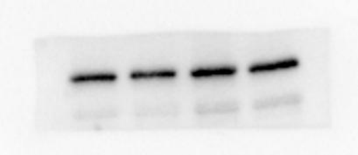 65KD

GAPDH 35KD 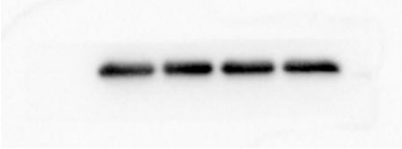 36KD

P-P65 50KD 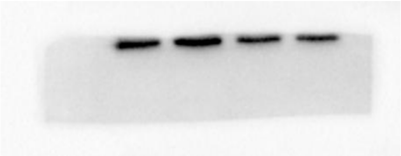 65KD

GAPDH 35KD

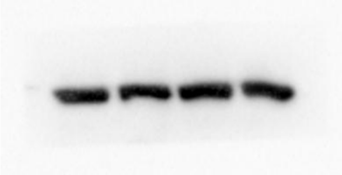

36KD

Repeat three：

P65 50KD

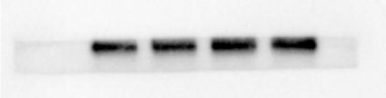

65KD

GAPDH 35KD

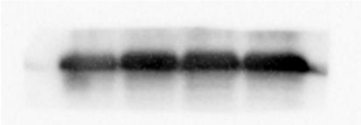

36KD

P-P65 50KD

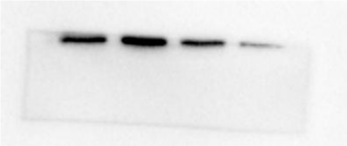

65KD

GAPDH 35KD

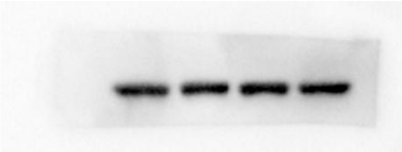

36KD

Repeat four：

P65

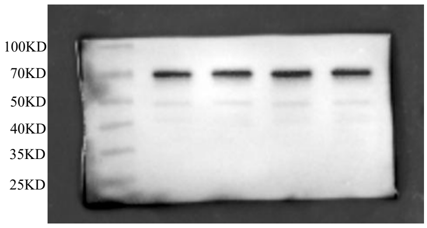

65KD

GAPDH

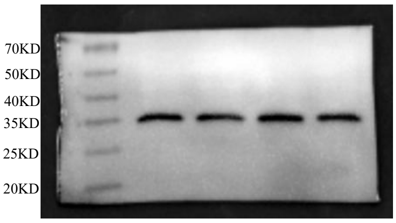

36KD

P-P65

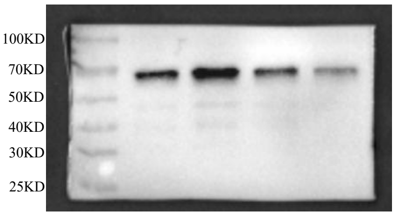

65KD

GAPDH

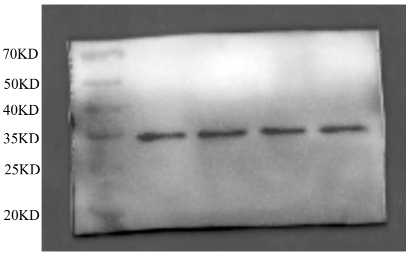

36KD

6、Figure 8C

Repeat one：

P65

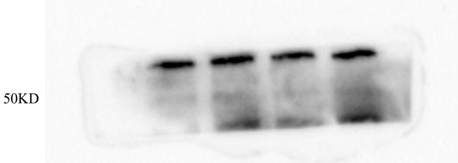

65KD

GAPDH

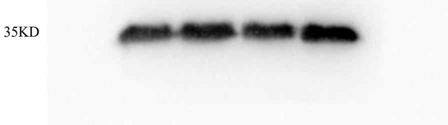

36KD

P-P65

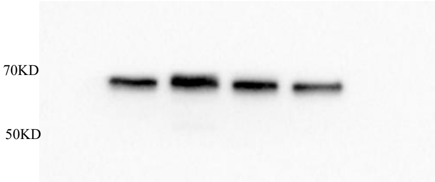

65KD

GAPDH

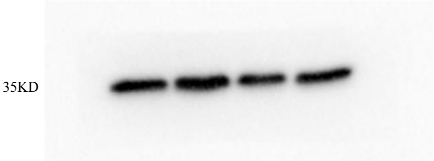

36KD

Repeat two：

P65

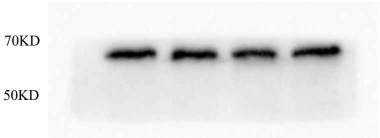

65KD

GAPDH

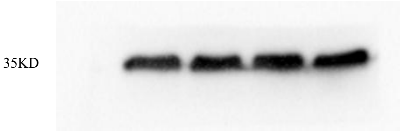

36KD

P-P65

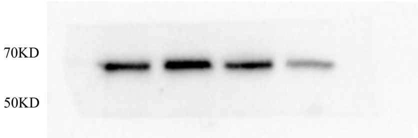

65KD

GAPDH

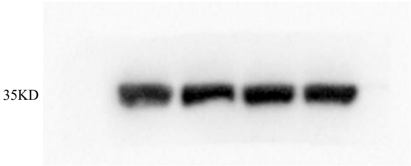

36KD

Repeat three：

P65

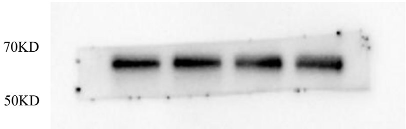

65KD

GAPDH

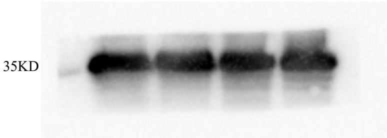

36KD

P-P65

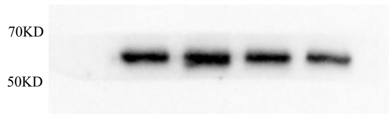

65KD

GAPDH

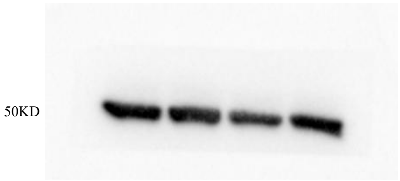

36KD

Repeat four：

P65

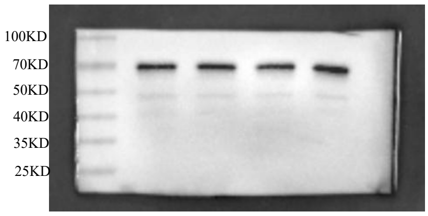

65KD

GAPDH

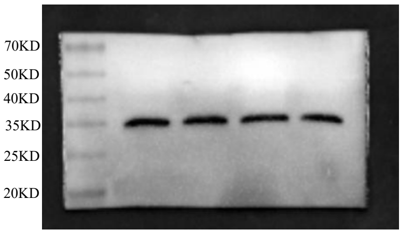

36KD

P-P65

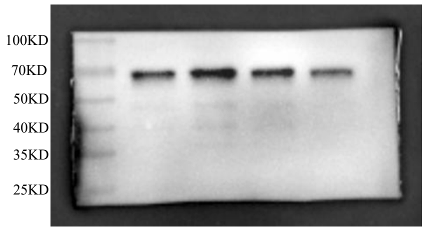

65KD

GAPDH

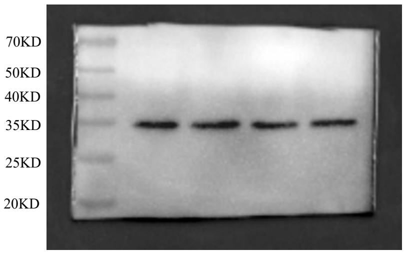

36KD

7、Figure 8E

Repeat one：

P65 70KD 50KD

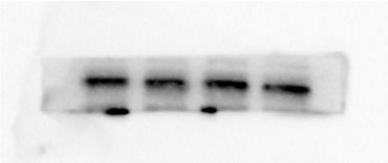

65KD

GAPDH 35KD

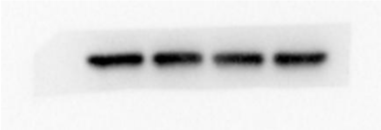

36KD

P-P65 70KD 50KD

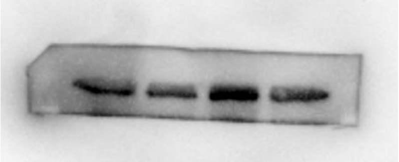

65KD

GAPDH 35KD

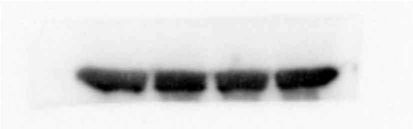

36KD

Repeat two：

P65 70KD 50KD

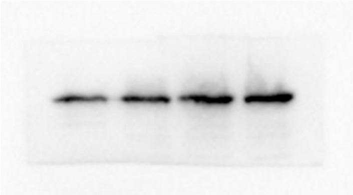

65KD

GAPDH 35KD

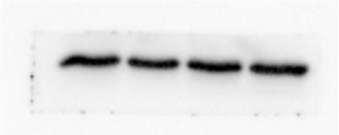

36KD

P-P65 70KD 50KD

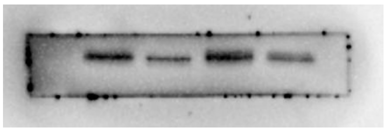

65KD

GAPDH 35KD

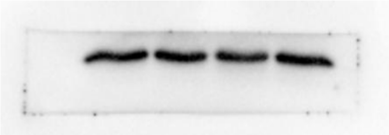

36KD

Repeat three：

P65 70KD 50KD

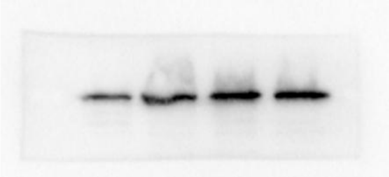

65KD

GAPDH 35KD

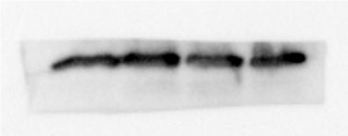

36KD

P-P65 70KD 50KD

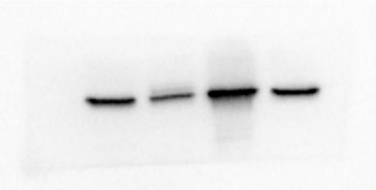

65KD

GAPDH 35KD

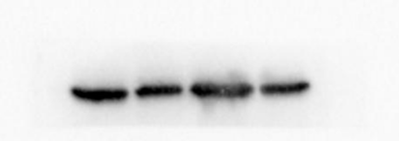

36KD

Repeat four：

P65 100KD 70KD 50KD 40KD 35KD 25KD

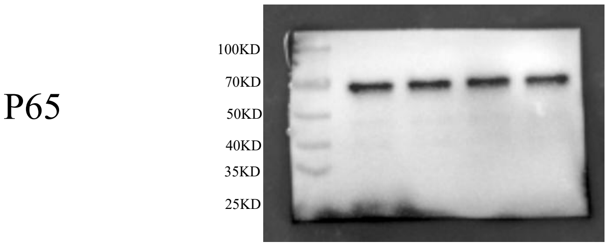

65KD

GAPDH 100KD 70KD 50KD 40KD 35KD 25KD

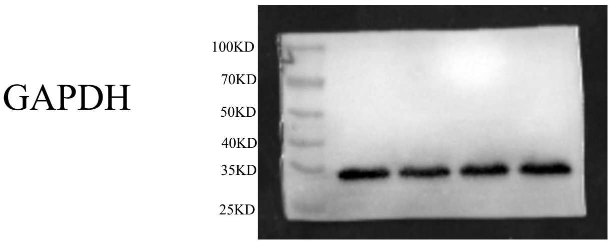

36KD

P-P65

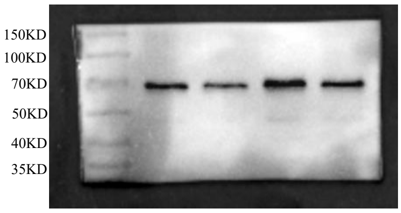

65KD

GAPDH

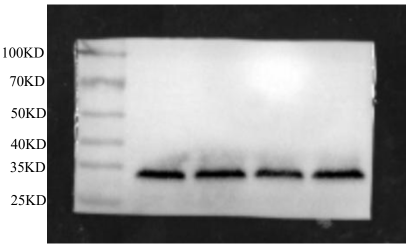

36KD

8、Figure 8G

Repeat one：

P65

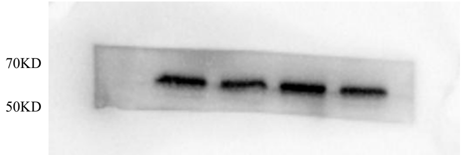

65KD

GAPDH

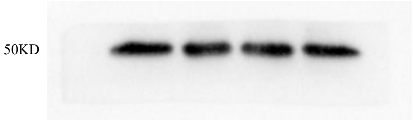

36KD

P-P65

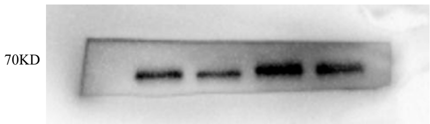

65KD

GAPDH

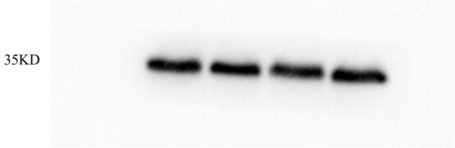

36KD

Repeat two：

P65

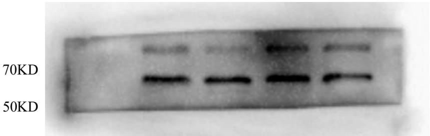

65KD

GAPDH 35KD

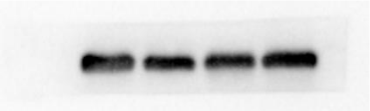

36KD

P-P65 70KD 50KD

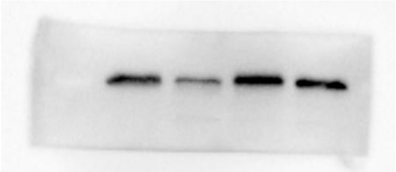

65KD

GAPDH 35KD

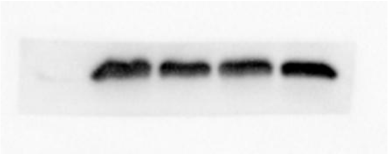

36KD

Repeat three：

P65 70KD 50KD

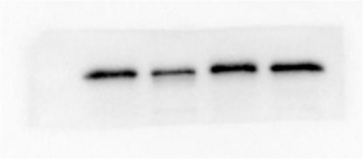

65KD

GAPDH 35KD

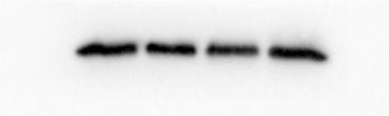

36KD

P-P65 70KD 50KD

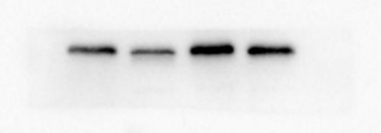

65KD

GAPDH 35KD

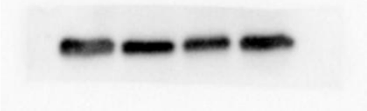

36KD

Repeat four：

P65 100KD 70KD 50KD 40KD 35KD 25KD

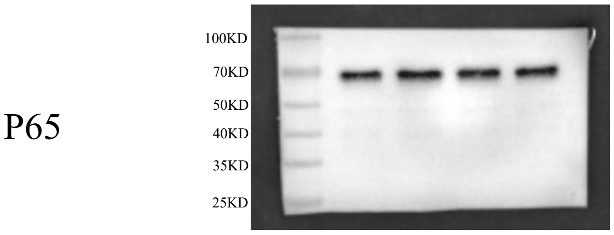

65KD

GAPDH 36KD

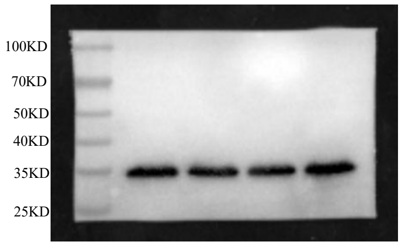

P-P65 65KD

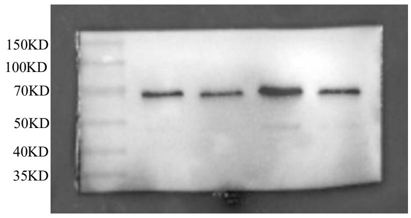

GAPDH 36KD

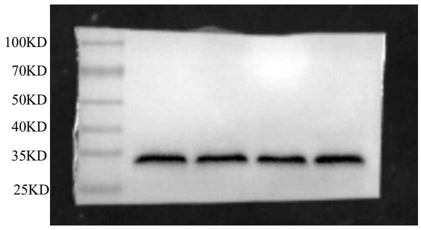

9、Figure 8I

Repeat one：

P65 65KD

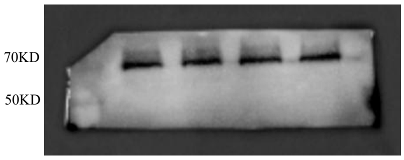

GAPDH 36KD

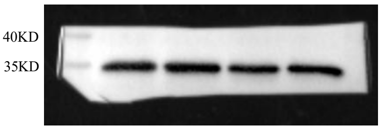

P-P65 65KD

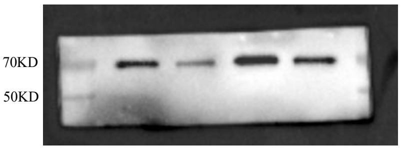

GAPDH 36KD

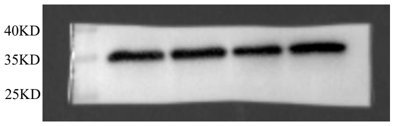

Repeat two：

P65

70KD
50KD

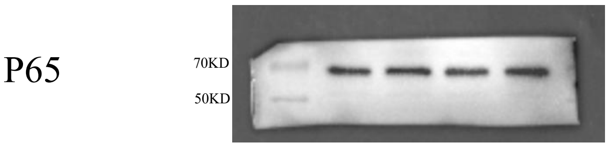

65KD

GAPDH

40KD
35KD

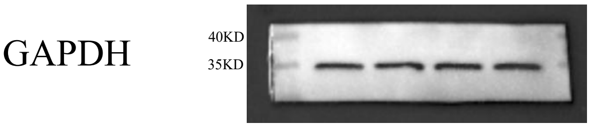

36KD

P-P65

70KD
50KD

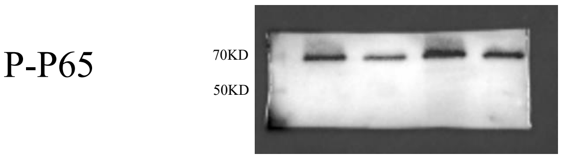

65KD

GAPDH

35KD

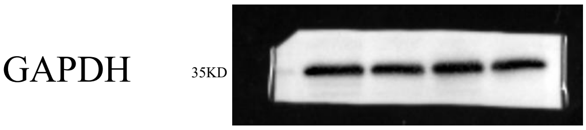

36KD

Repeat three：

P65

70KD
50KD

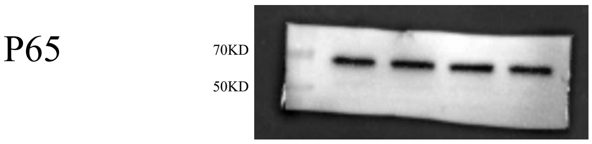

65KD

GAPDH

40KD
35KD

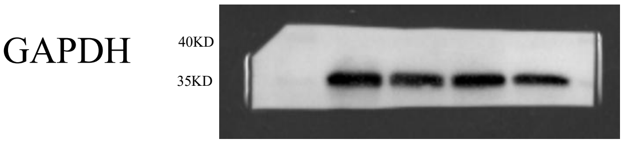

36KD

P-P65

70KD
50KD

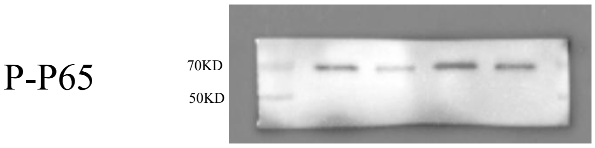

65KD

GAPDH

40KD
35KD

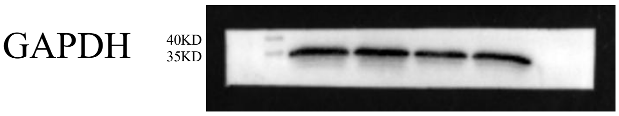

36KD

Repeat four：

P65

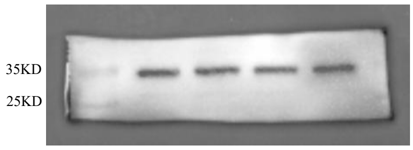

65KD

GAPDH

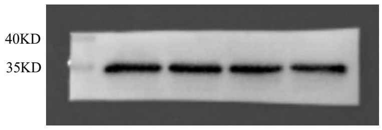

36KD

P-P65

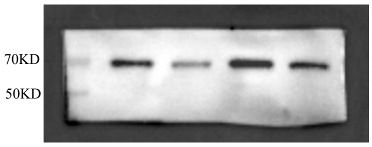

65KD

GAPDH

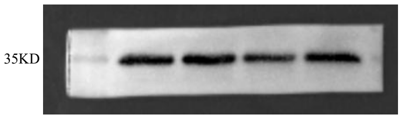

36KD

Repeat five：

P65

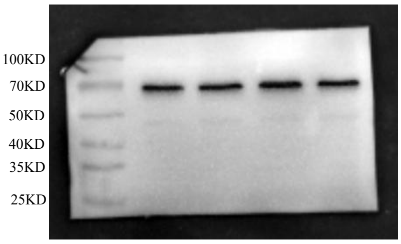

65KD

GAPDH

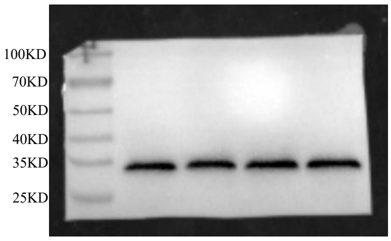

36KD

P-P65

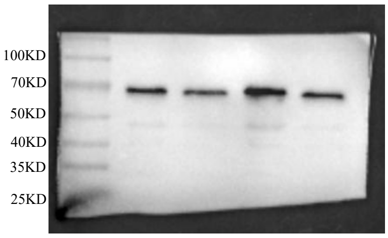

65KD

GAPDH 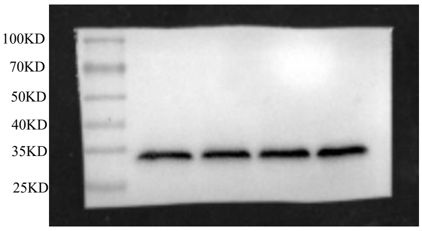

36KD

10、Supplementary figure 1A

Repeat one：

Input: NCAPH-FLAG 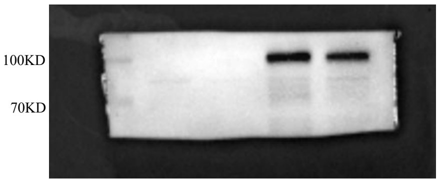

82KD

Input: IKB-HIS 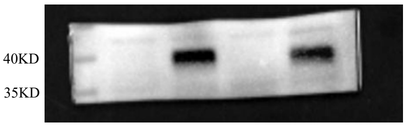

40KD

Input: GAPDH 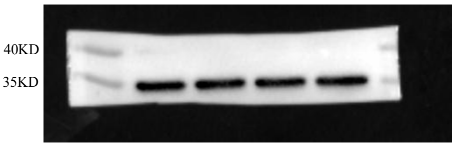

36KD

IP: NCAPH-FLAG 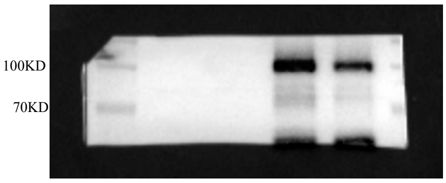

82KD

IP: IKB-HIS 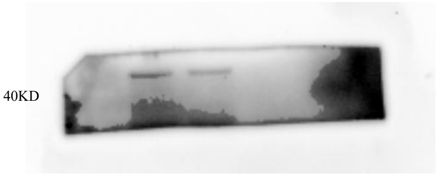

40KD

Repeat two：

Input: NCAPH-FLAG

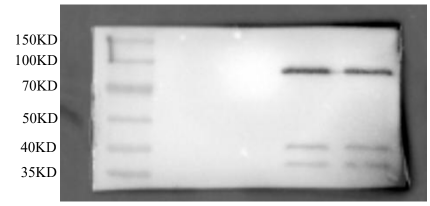

82KD

Input: IKB-HIS

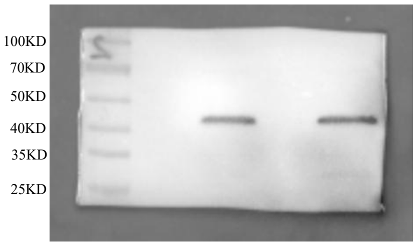

40KD

Input: GAPDH

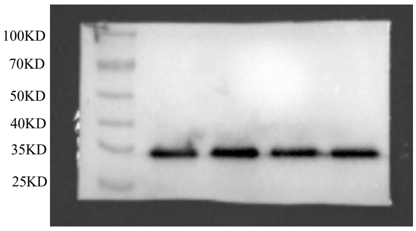

36KD

IP: NCAPH-FLAG

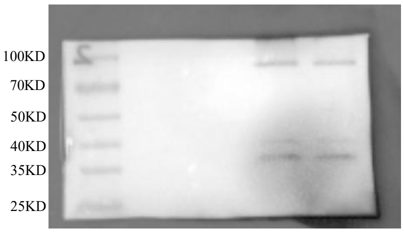

82KD

IP: IKB-HIS

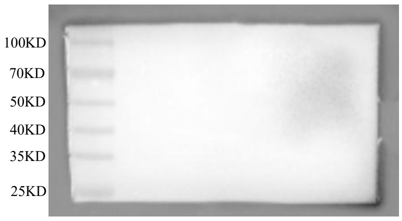

40KD

11、Supplementary figure 1B

Repeat one：

Input: NCAPH-FLAG

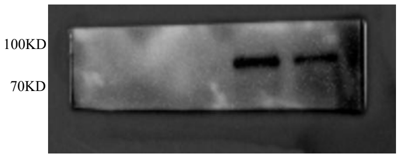

82KD

Input: IKB-HIS

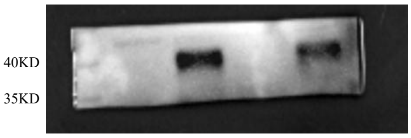

40KD

Input: GAPDH

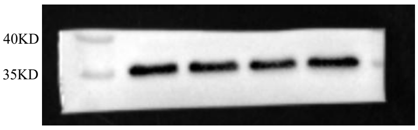

36KD

IP: NCAPH-FLAG

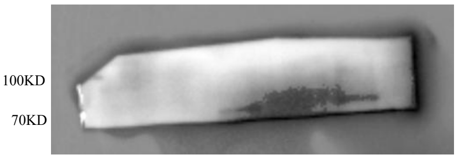

82KD

IP: IKB-HIS

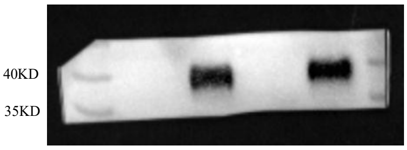

40KD

Repeat two：

Input: NCAPH-FLAG

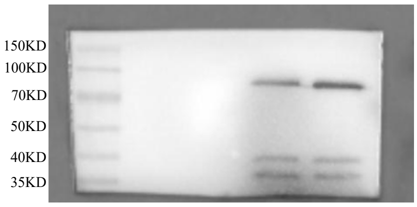

82KD

Input: IKB-HIS

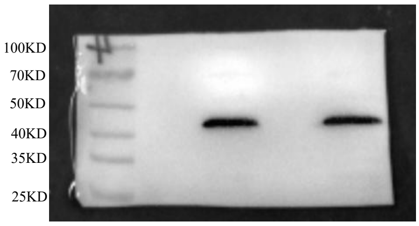

40KD

Input: GAPDH

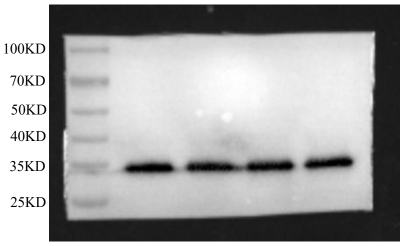

36KD

IP: NCAPH-FLAG

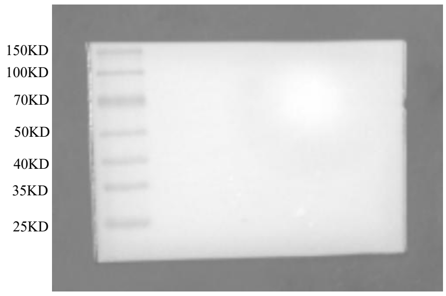

82KD

IP: IKB-HIS

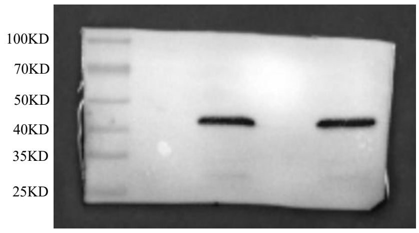

40KD

12、Supplementary figure 1C

Repeat one：

NCAPH

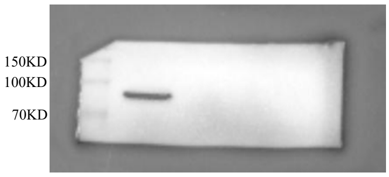

82KD

P65

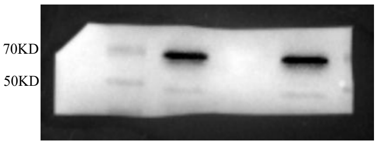

65KD

Repeat two：

NCAPH

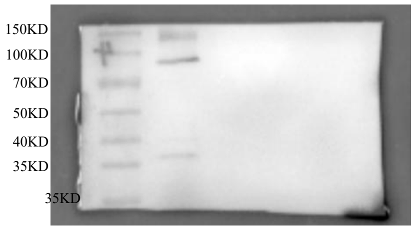

82KD

P65

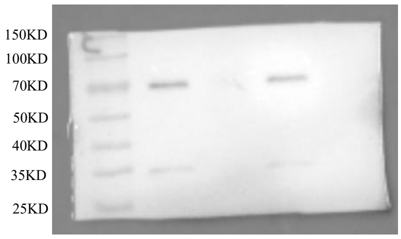

65KD

13、Supplementary figure 1D

Repeat one：

NCAPH

150KD
100KD
70KD

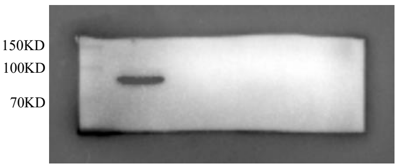

82KD

P65

70KD
50KD
40KD

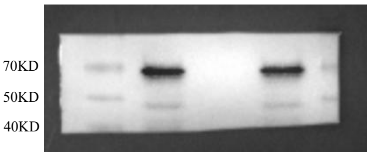

65KD

Repeat two：

NCAPH

150KD
100KD
70KD
50KD
40KD
35KD

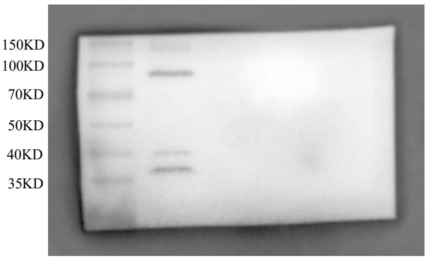

82KD

P65

100KD
70KD
50KD
40KD
35KD
25KD

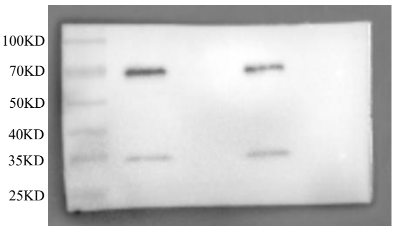

65KD
